# Coffee consumption is associated with intestinal *Lawsonibacter asaccharolyticus* abundance and prevalence across multiple cohorts

**DOI:** 10.1038/s41564-024-01858-9

**Published:** 2024-11-18

**Authors:** Paolo Manghi, Amrisha Bhosle, Kai Wang, Roberta Marconi, Marta Selma-Royo, Liviana Ricci, Francesco Asnicar, Davide Golzato, Wenjie Ma, Dong Hang, Kelsey N. Thompson, Eric A. Franzosa, Amir Nabinejad, Sabrina Tamburini, Eric B. Rimm, Wendy S. Garrett, Qi Sun, Andrew T. Chan, Mireia Valles-Colomer, Manimozhiyan Arumugam, Kate M. Bermingham, Francesca Giordano, Richard Davies, George Hadjigeorgiou, Jonathan Wolf, Till Strowig, Sarah E. Berry, Curtis Huttenhower, Tim D. Spector, Nicola Segata, Mingyang Song

**Affiliations:** 1 Department CIBIO, University of Trento, Trento, Italy; 2 Harvard T.H. Chan School of Public Health, Boston, MA USA; 3 The Broad Institute of MIT and Harvard, Cambridge, MA USA; 4 Massachusetts General Hospital and Harvard Medical School, Boston, MA USA; 5 IEO, Istituto Europeo di Oncologia IRCSS, Milan, Italy; 6 Department of Molecular Sciences and Nanosystems, Ca’ Foscari University, Venice, Italy; 7 Harvard Chan Microbiome in Public Health Center, Boston, MA USA; 8 Brigham and Women’s Hospital and Harvard Medical School, Boston, MA USA; 9 MELIS Department, University Pompeu Fabra, Barcelona, Spain; 10 Novo Nordisk Foundation Center for Basic Metabolic Research, University of Copenhagen, Copenhagen, Denmark; 11 Department of Nutritional Sciences, King’s College London, London, UK; 12 ZOE Ltd, London, UK; 13 Helmholtz Center for Infection Research, Braunschweig, Germany; 14 Department of Twins Research and Genetic Epidemiology, King’s College London, London, UK; 15 Present Address: Computational Biology Unit, Research and Innovation Centre, Fondazione Edmund Mach, San Michele all’Adige, Italy

**Keywords:** Epidemiology, Microbiome

## Abstract

Although diet is a substantial determinant of the human gut microbiome, the interplay between specific foods and microbial community structure remains poorly understood. Coffee is a habitually consumed beverage with established metabolic and health benefits. We previously found that coffee is, among >150 items, the food showing the highest correlation with microbiome components. Here we conducted a multi-cohort, multi-omic analysis of US and UK populations with detailed dietary information from a total of 22,867 participants, which we then integrated with public data from 211 cohorts (*N* = 54,198). The link between coffee consumption and microbiome was highly reproducible across different populations (area under the curve of 0.89), largely driven by the presence and abundance of the species *Lawsonibacter asaccharolyticus*. Using in vitro experiments, we show that coffee can stimulate growth of *L.* *asaccharolyticus*. Plasma metabolomics on 438 samples identified several metabolites enriched among coffee consumers, with quinic acid and its potential derivatives associated with coffee and *L.* *asaccharolyticus*. This study reveals a metabolic link between a specific gut microorganism and a specific food item, providing a framework for the understanding of microbial dietary responses at the biochemical level.

## Abstract (web summary)

Coffee consumption is associated with the presence and abundance of a specific member of the human gut microbiome, *Lawsonibacter asaccharolyticus*, and changes to the plasma metabolome.

## Main

Coffee is consumed almost worldwide and has been shown to exert beneficial effects on human health, including lowering all-cause and cardiovascular disease-specific mortality^1,2^, risks of type 2 diabetes^3,4^, non-alcoholic fatty liver disease^5^ and cancer^6,7^, as well as other diseases^8,9^. Coffee’s nutritional epidemiology is unique in that it is typically either consumed every day or not consumed at all and is thus reported with high accuracy^10^. Combined with its unique chemical composition, this makes coffee an excellent model to unravel the metabolomic processes by which the gut microbiome responds to dietary components.

The benefits of coffee may be ascribable to some of its polyphenol components including chlorogenic acid, the caffeic ester of quinic acid, and *N*-methylpyridium, a derivative of trigonelline^9,11–20^. The gut microbiome is involved in the metabolism of coffee and potentially mediates its health effects^11,12,16,21–25^. One small study showed an increase in *Bacteroides*, *Porphyromonas* and *Prevotella* among coffee drinkers in a cohort of 147 healthy individuals using quantitative PCR^22^. A 16S ribosomal RNA gene amplicon sequencing study on the effects of coffee on murine gut microbial communities also reported an increase in *Prevotella*^16^. Similarly, 16S rRNA gene amplicon sequencing of the colonic mucosal microbiomes from 34 healthy participants showed higher alpha diversity and an increase in the genera *Faecalibacterium* and *Alistipes* in response to caffeine^23^. In support of the uniqueness of coffee’s interaction with the gut microbiome, our initial ZOE Personalized Responses to Dietary Composition Trial (PREDICT 1) metagenomics study^25^ showed that coffee consumption had, among over 150 food items, the highest correlation with gut microbiome composition in ~1,000 individuals.

In this study, we conducted the largest investigation so far of the human gut microbiome link with coffee consumption by leveraging the ZOE PREDICT cohorts, the Mind–Body Study (MBS) and the Men’s Lifestyle Validation Study (MLVS). Together, we analysed over 22,000 shotgun metagenomic samples from participants who provided detailed reports on long-term coffee consumption. This showed a tight interplay between coffee and a specific microbiome member, the *Lawsonibacter asaccharolyticus* species, which was recently isolated from human faeces^26^. We verified that it grows better in vitro when media is supplemented with coffee. By analysing joint plasma metabolomes, we showed a *L.* *asaccharolyticus*-dependent increase in blood levels of quinic acid, trigonelline and caffeine, plus other uncharacterized molecules, among coffee drinkers compared with non-drinkers. This work helps unravel the role of the human microbiome in coffee metabolism and shows the potential for future microbiome studies of individual dietary factors.

## Results

### Studying the interplay between coffee and the microbiome

**Fig. 1:**
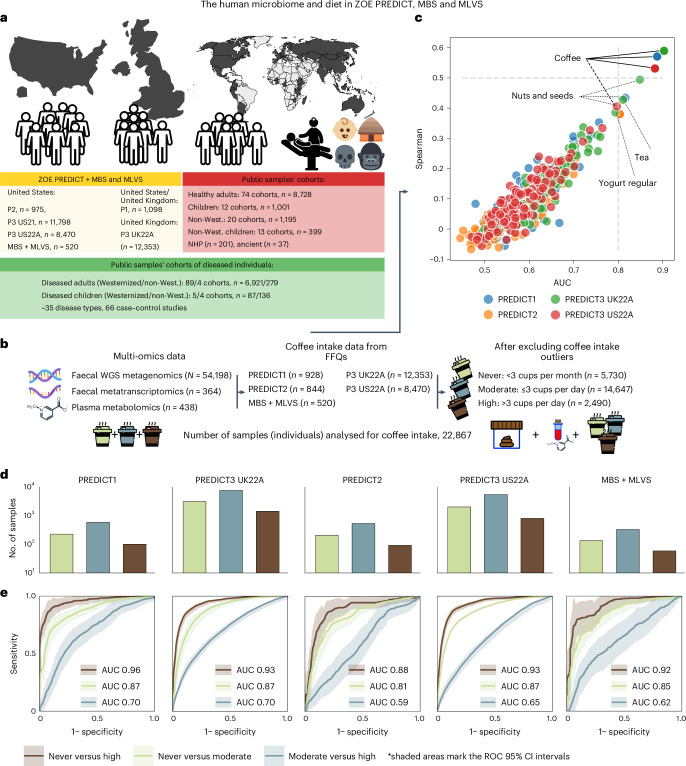
Consistent global links between coffee consumption and the human gut microbiome. **a**, Five UK and/or US PREDICT cohorts (*n* = 975, 11,798, 8,470, 1,098 and 12,353), the MBS and the MLVS (*n* = 213 and *n* = 307, respectively) were used to assess diet–microbiome relationships (total *n* = 35,214). For later comparisons of microbiome distributions across different populations, we retrieved *n* = 18,984 metagenomic samples from public sources, including healthy adult individuals, newborns, non-Westernized (non-West.) individuals, ancient samples and non-human primates (NHP). P1, PREDICT1; P2, PREDICT2; P3, PREDICT3. **b**, We combined faecal metagenomics (*n* = 54,198), faecal metatranscriptomics (*n* = 364) and plasma metabolomics (*n* = 438), with the latter two from the MBS and MLVS cohorts. FFQs surveyed nutritional habits of the participants from four PREDICT cohorts, MBS and MLVS (*n* = 22,867 after removing individuals above the 99th percentile of coffee intake in the PREDICT cohorts as outliers). Participants were categorized as ‘high’, ‘moderate’ and ‘never’ coffee drinkers as previously established^25^. **c**, Median Spearman’s correlation and median AUCs from a random forest regressor and a random forest classifier trained on the microbiome composition estimated by MetaPhlAn 4 (ref. ^30^). **d**, The number of never (light green), moderate (dark cyan) and high-coffee drinkers (brown). **e**, ROC and AUC of random forest classifiers discriminating participants between pairs of the three coffee drinker classes, assessed in a tenfold, ten times repeated cross-validations (CV) that benefited from the other cohorts during the training phase as in the leave-one-dataset-out approach (LODO; Methods). The shaded areas represent the 95% confidence intervals (CIs) of a linear interpolation over all the folds of the test. Machine learning results using either only a CV or a LODO approach are reported in Extended Data Fig. 2a,b.

A total of 35,214 metagenomes from five ZOE PREDICT cohorts (PREDICT1, PREDICT2, PREDICT3 US21, PREDICT3 US22A and PREDICT3 UK22A)^27^, the MBS^28^, and the MLVS^29^ were included in this study, together with 18,984 metagenomes from public sources including healthy individuals (*n* = 8,728), non-Westernized individuals (*n* = 1,195; Methods), newborns and infants (*n* = 1,623), ancient microbiome samples (*n* = 37), non-human primates (*n* = 201) and 7,423 samples from individuals with a specific disease (35 diseases). Overall, these sets lead to a total number of 54,198 metagenomic samples considered (Fig. 1a), all profiled using MetaPhlAn 4 (ref. ^30^). In addition, the metagenomic profiles of 23,115 of these samples were coupled with highly detailed food frequency questionnaires (FFQs) covering over 150 food items from the PREDICT1, PREDICT2, PREDICT3 US22A, PREDICT3 UK22A, MBS and MLVS cohorts (Methods). We also analysed 438 plasma metabolomes and 364 faecal metatranscriptomes from the MBS and MLVS cohorts (Fig. 1b).

Taking advantage of the four PREDICT cohorts with full dietary information (*n* = 22,595), we first used a random forest algorithm to associate single food items with microbiome. Microbiome species-level genome bin (SGB) composition was highly predictive of total coffee intake from FFQs in a cross-validation setting. Spearman’s correlation between predicted versus real coffee consumption reached values above 0.5 for the PREDICT1, PREDICT3 US22A and PREDICT3 UK22A studies. When using random forests to distinguish the top versus bottom quartiles of food intake, coffee was again the one most accurately predicted by microbiome composition (area under the curve (AUC) of >0.8; Fig. 1c). Importantly, milk, dairy cream, sugar and honey, which are commonly added to coffee and are present in the PREDICT FFQs, achieved much lower prediction performances (Spearman’s *ρ* < 0.1, AUC ~0.55–0.58), with the only partial exception of milk and soy/rice milk in the PREDICT3 UK22A cohort (*ρ* = 0.26 and 0.21, AUC of 0.76 and 0.7, respectively). Coffee is therefore not only a food with ascertained health effects, but also most strongly associated with the human microbiome among ~150 food items.

### Coffee consumption is strongly linked to the gut microbiome

**Extended Data Fig. 1:**
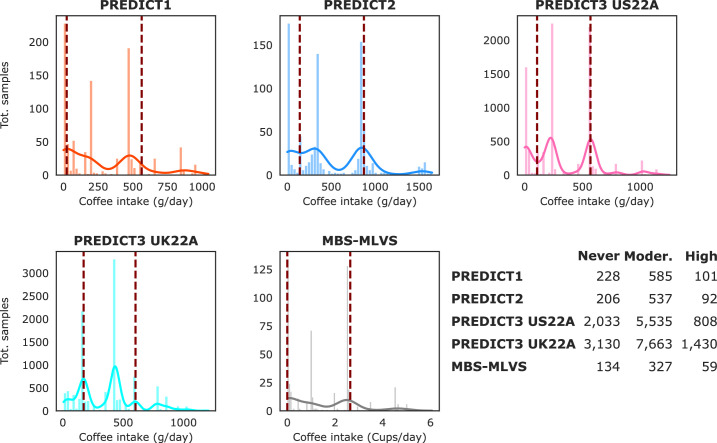
Coffee intake estimated via FFQ in four ZOE PREDICT and the MBS-MLVS cohorts. X-axis shows the estimated grams per day (ZOE PREDICT) and cups per day (MBS-MLVS), Y-axis shows the number of samples. Brown dashed lines indicate the 24.94^th^ and 88.95^th^ percentiles that correspond to the thresholds of 20 and 600 grams per day in the PREDICT1 cohort and have been used to determine the three categories never, moderate, and high coffee drinkers in the five cohorts. Continuous darker line marks the Gaussian kernel density estimation. Number of microbiome samples per cohort per category are also reported.

To better characterize the link between the gut microbiome and coffee, we classified individuals from PREDICT1, PREDICT2, PREDICT3 US22A, PREDICT3 UK22A and MBS–MLVS into three coffee-drinking levels: ‘never’, ‘moderate’ and ‘high’ (Fig. 1b,d). This categorization used the same thresholds previously applied by a panel of nutritionists in the PREDICT1 study^25^. Individuals with a coffee intake up to 20 g a day (less than three cups a month) were classified into the group ‘never’, individuals with an intake ≥600 g of coffee per day (more than three cups a day) were classified as ‘high’ and coffee drinkers between these two values were categorized as ‘moderate’. These thresholds, representing the 24.9th and 88.95th percentiles of coffee intake in the PREDICT1 cohort (Supplementary Table 1), were applied to all other cohorts (Fig. 1d and Extended Data Fig. 1). In addition, samples from individuals with a coffee intake above the 99th percentile were excluded as outliers potentially due to data collection issues. In total, 5,730 individuals were classified as ‘never’, 14,647 as ‘moderate’ and 2,490 as ‘high’ coffee drinkers (Fig. 1d and Supplementary Table 2).

**Extended Data Fig. 2:**
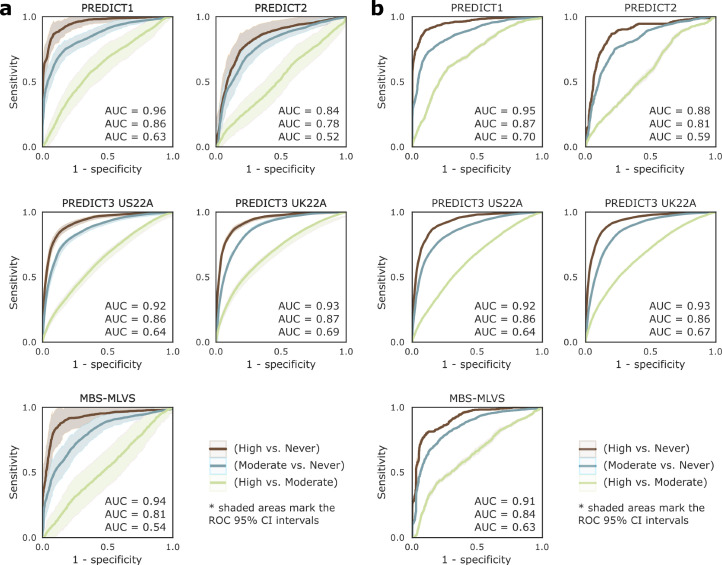
Cross validation and Leave-one-dataset-out validation. **a)** receiver operating characteristic (ROC) curves and areas under the curve (AUCs) of random forest algorithms discriminating participants of the three combinations: never vs. moderate (light green), moderate vs. high (dark cyan), and high vs. never (brown), using the microbiome composition estimated by MetaPhlAn 4, assessed in a ten-fold, ten-times repeated cross-validations. **b)** ROC curve and AUCs of the same algorithm and experiment, using a leave-one-dataset-set-out (LODO) approach. Shaded areas represent the 95% confidence intervals of a linear interpolation over all the folds of the test.

We next used machine learning to evaluate the strength of the link between microbiome composition and coffee consumption levels^31,32^. We employed random forest classifiers trained on SGB-level abundances to distinguish between three pairs of conditions: never versus moderate, moderate versus high and never versus high coffee drinkers. The high and moderate categories were both highly separable from the never category (tenfold, ten times repeated cross-validation, median AUC across cohorts of 0.92 and 0.86, respectively; Extended Data Fig. 2a and Supplementary Table 3). Weaker predictions were achieved for moderate versus high coffee drinkers (median AUC of 0.63), suggesting a limited dose-dependent association of coffee intake with the microbiome.

Cross-validation performances in distinguishing coffee consumption levels were consistent across datasets, but to further test the cross-population reproducibility of the microbiome signature, we applied the leave-one-dataset-out (LODO)^32^ as well as a ‘cross-LODO’ hybrid approach (that is, augmenting the training folds of a specific dataset with external datasets; Methods). The ‘cross-LODO’ analysis yielded a median AUC of 0.93 for the never versus high comparison, 0.87 for the never versus moderate comparison and 0.65 for the moderate versus high comparison (Fig. 1e, Extended Data Fig. 2b and Supplementary Table 3). These findings suggest that the gut microbiome has distinct compositions in coffee drinkers compared with non-coffee drinkers, with a modest effect on differentiating the dose of coffee drinking.

### *L. asaccharolyticus* is strongly linked with coffee

**Extended Data Fig. 3:**
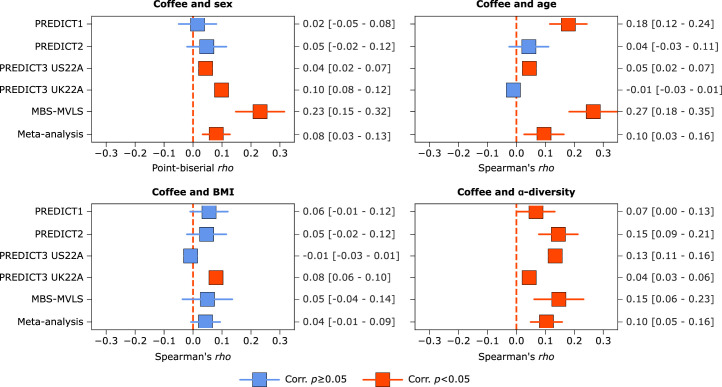
Microbiome-correlated variables, sex, age, BMI, and ɑ-diversity are correlated with coffee intake. Point-biserial correlation between sex and coffee, and Spearman’s correlation between age, BMI, ɑ-diversity and coffee consumption. Correlations coefficients are then pooled in a random-effects meta-analysis. Blue marks single study or pooled correlation which *p* ≥ 0.05, red marks single/pooled correlations which *p* < 0.05.

To investigate which gut microbiome features are associated with coffee intake, we correlated the ranked abundances of SGBs with participant’s coffee intake in each cohort (Spearman’s correlation) using raw and partial correlations (Supplementary Table 4 and Methods). Correlations were then meta-analysed across all cohorts (Supplementary Table 5 and Methods). In total, we found 291 correlations at *q* < 0.001 with 132 SGBs having *ρ* ≥ 0.05, and 298 when taking into account the effect of sex, age and body mass index (BMI) of the participants (*q* < 0.001, 130 with *ρ* ≥ 0.05; Supplementary Tables 6 and 7 and Extended Data Fig. 3).

**Fig. 2:**
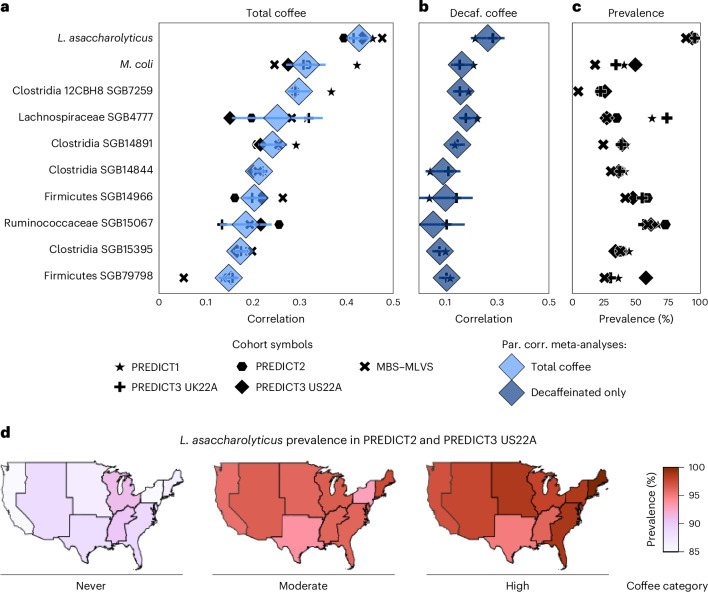
*L.* *asaccharolyticus* drives the association between the gut microbiome and coffee intake. **a**, The top ten SGBs from a meta-analysis of partial correlations between SGB-ranked abundances and total per-individual coffee intake considering the five cohorts analysed in this study (*q* < 0.001). The black markers show the per-cohort partial correlations and the light blue markers indicate the average Spearman’s correlations adjusted by sex, age and BMI. **b**, The same SGBs are meta-analysed with Spearman’s partial correlations (par. corr.) between SGB abundances and decaffeinated (decaf.) coffee intake in the PREDICT1 and PREDICT3 UK22A cohorts, excluding individuals who consumed caffeinated coffee only (*n* = 262 and 4,055). The black markers show the per-cohort correlations and dark blue symbols refer to average correlations adjusted by sex, age, BMI and caffeinated coffee. **c**, The prevalence of the ten SGBs in the five cohorts analysed. **d**, The prevalence of *L.* *asaccharolyticus* across never, moderate and high coffee drinkers and nine US regions in the PREDICT2 and PREDICT3 US22A cohorts (*n* = 9,210).

**Extended Data Fig. 4:**
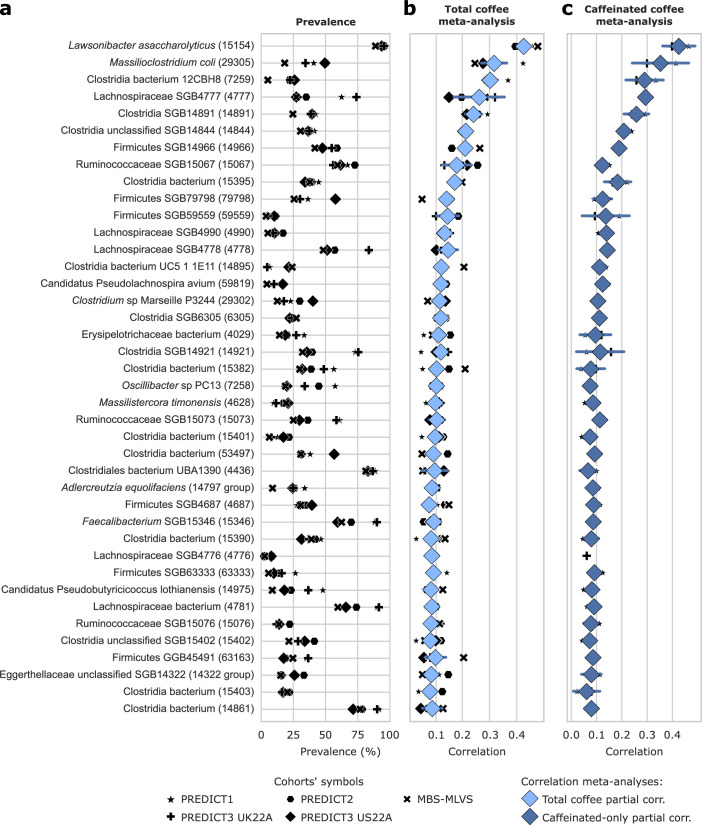
Meta-analysis of rank correlation between SGBs and coffee intake. **a)** Prevalences in the five cohorts considered in this study of the 40 strongest SGB-abundance and coffee-intake pooled partial Spearman’s correlations found at *q* < 0.001. **b)** Single-study partial Spearman’s correlation between SGB relative abundance and per-individuals total coffee intake, adjusting by age, sex, and BMI (black symbols) and pooled partial correlation (light blue markers). **c)** Single-study partial Spearman’s correlation between SGB relative abundance and per-individuals caffeinated coffee intake only (black symbols), adjusting by age, sex, BMI, and decaffeinated coffee intake and pooled partial correlation (dark blue markers). Only the PREDICT1 and PREDICT3 22UKA cohorts were used in this analysis.

While most of the correlations were positive, 46 partial correlations were negative and significant (*q* < 0.001), although none exceeded *ρ* values lower than −0.1. This suggests more stimulatory rather than inhibitory effects of coffee and its components on microbial species relative abundances (Extended Data Fig. 4a,b). Among the positive correlations, the strongest one involved the species *L.* *asaccharolyticus* (SGB15154), reaching *ρ* = 0.43 (0.41–0.45) and *q* < 10^−10^. In contrast, correlation between coffee and ɑ-diversity was much lower (*ρ* = 0.1 (0.05–0.15), *p* = 1.8 × 10^−4^; Extended Data Fig. 3). *L.* *asaccharolyticus* was first isolated in 2018^26,33^, and as this strain remains the only one deposited in public biobanks, here we consider it to be representative for the species although other genomes (but not isolates) with conflicting taxonomic labels have been deposited (that is, *Clostridium phoceensis*^34^; Methods). The species responsible for the next two strongest associations were *Massilioclostridium coli* (SGB29305, *ρ* = 0.31 (0.27–0.35), *q* < 10^−10^) and the so-far uncharacterized *Clostridium* species ‘12CBH8’ (SGB7259, *ρ* = 0.3 (0.28–0.32), *q* < 10^−10^; Fig. 2a).

We further investigated whether these associations were driven by caffeine by performing two meta-analyses on the PREDICT1 and PREDICT3 22UKA samples for which the intake of decaffeinated and caffeinated coffee was available. Partial correlations between SGB-ranked abundances and decaffeinated versus caffeinated coffee were run independently (excluding individuals who drank exclusively caffeinated or decaffeinated coffee, respectively). In addition, partial correlations were also adjusted for the other type of coffee consumed by the individuals in case their record reported both kinds. We identified 150 correlations, which remained highly significant after controlling for the decaffeinated coffee intake (*q* < 0.001, *n*_participants_ = 12,089; Extended Data Fig. 4c and Supplementary Table 8). This indicated a substantial independence on caffeine of the observed impact on the microbiome. Next, we analysed the decaffeinated coffee association with the microbiome in individuals consuming decaffeinated coffee and adjusting by caffeinated coffee as well as by sex, age and BMI. In this reduced set of samples (*n*_participants_ = 6,089) we identified 22 correlations at *q* < 0.001 and 66 at *q* < 0.1 (Supplementary Table 9). The top three correlations identified were *L.* *asaccharolyticus* (*ρ* = 0.27 (0.21–0.33), *q* < 10^−10^), the Lachnospiraceae SGB4777 (*ρ* = 0.18 (0.16–0.21), *q* < 10^−10^), and *M.* *coli* (SGB29305, *ρ* = 0.17 (0.13–0.2), *q* < 10^−10^; Fig. 2b).

**Extended Data Fig. 5:**
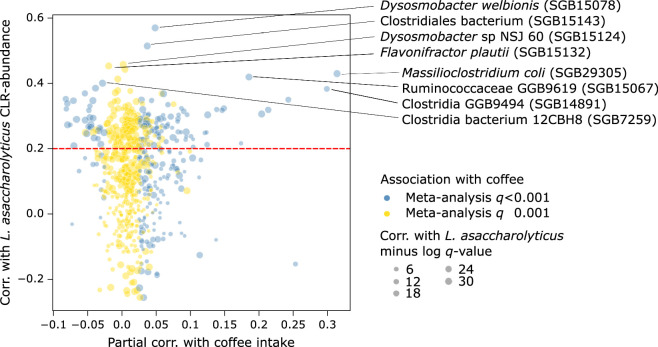
Coffee-associated SGBs tend to be correlated in abundance with *L. asaccharolyticus.* (X-axis) correlation coefficients from the partial correlation meta-analysis with coffee consumption (Supplementary Table 7) vs correlation coefficients from a correlation meta-analysis with *L. asaccharolyticus* abundance (Y-axis, Supplementary Table 10). Correlations between *L. asaccharolyticus* and the other SGBs (Spearman’s) were computed after centred log-ratio transformation (CLR) following zero imputation with a multiplicative replacement method to control for the problem of relative abundances’ correlated structure. SGBs below 10% prevalence and not present in at least two cohorts were excluded (in total 707 SGBs were evaluated).

As expected, several coffee-associated SGBs were also *L.* *asaccharolyticus* co-abundant SGBs, possibly indicating similar independent stimulatory effects of coffee rather than ecological relations (Extended Data Fig. 5 and Supplementary Table 10). The top-five SGBs associated with *L.* *asaccharolyticus* abundance were, however, not among the strongest associations with coffee. In particular, the two SGBs with the strongest co-abundance pattern with *L.* *asaccharolyticus* were *Dysosmobacter welbionis* (SGB15078) and the Clostridiales bacterium SGB15143 (*ρ* = 0.57 and 0.51, respectively, *q* < 1 × 10^−10^; Extended Data Fig. 5), which were both only weakly associated with total coffee (*ρ* ≤ 0.05; Supplementary Table 7). Overall, these results indicate that a panel of species, and in particular *L.* *asaccharolyticus* is robustly associated with total and decaffeinated coffee consumption, suggesting that the association is not purely due to caffeine.

### Effect of coffee on *L. asaccharolyticus* is supported in vitro

**Fig. 3:**
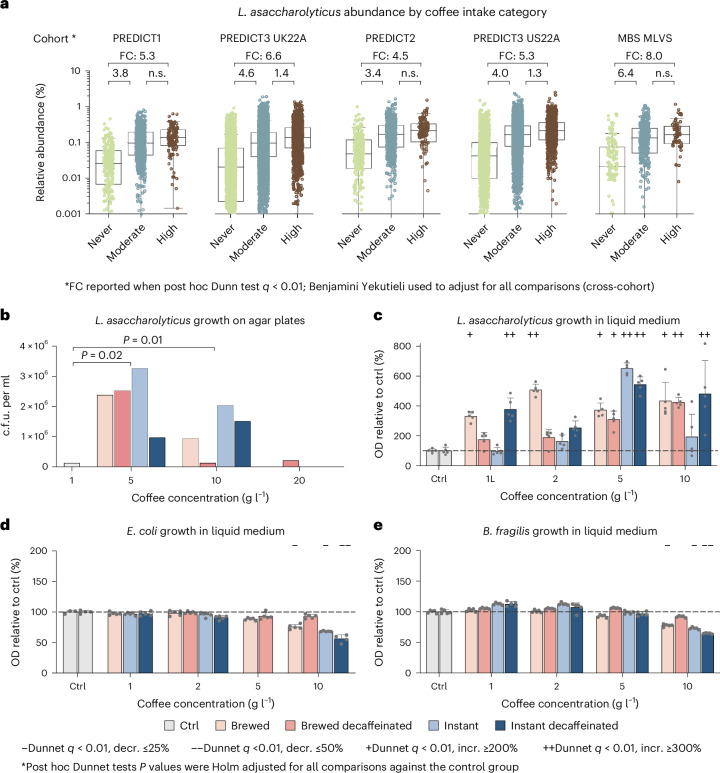
*L.* *asaccharolyticus* is highly prevalent with about fourfold higher average abundance in coffee drinkers, and its growth is stimulated by coffee supplementation in vitro*.* **a**, The relative abundance of *L.* *asaccharolyticus* in each cohort by coffee consumption category (never, moderate or high). The boxes represent the median and interquartile range (IQR) of the distributions, and top and bottom whiskers mark the point at 1.5 IQR. The median fold change of the high versus never comparison is reported on top if post hoc Dunn *q* < 0.01, and median fold change (FC) of the other two comparisons are reported on the top of each combination. n.s. (not significant) refers to post hoc Dunn *q* > 0.01. Total sample sizes are presented in Extended Data Fig. 1. **b**, *L.* *asaccharolyticus* growth on agar plates supplemented with increasing concentrations of coffee and measured by plate count (c.f.u. per ml). *P* values refer to one-sample *t*-tests compared with the control (ctrl) experiment value. **c**–**e**, Bacterial growth of *L.* *asaccharolyiticus* (**c**), *E.* *coli* (**d**) and *B.* *fragilis* (**e**) in liquid medium supplemented with increasing coffee concentrations and measured by changes in optical density (OD_650_). Percentage growth is relative to the culture medium control not supplemented with coffee (100%). Absolute OD_650_ values are reported in Supplementary Tables 15 and 16. The bars and error lines indicate the mean ± s.d. of five technical replicates, except for *E.* *coli* control (*n* = 3 and *n* = 4) and *B.* *fragilis* instant 5 g l^−1^ (*n* = 4). The minus and plus signs refer to significant tests (Dunnett *q* < 0.01) that overcome specific thresholds of fold increase (incr.) or decrease (decr.).

**Extended Data Fig. 6:**
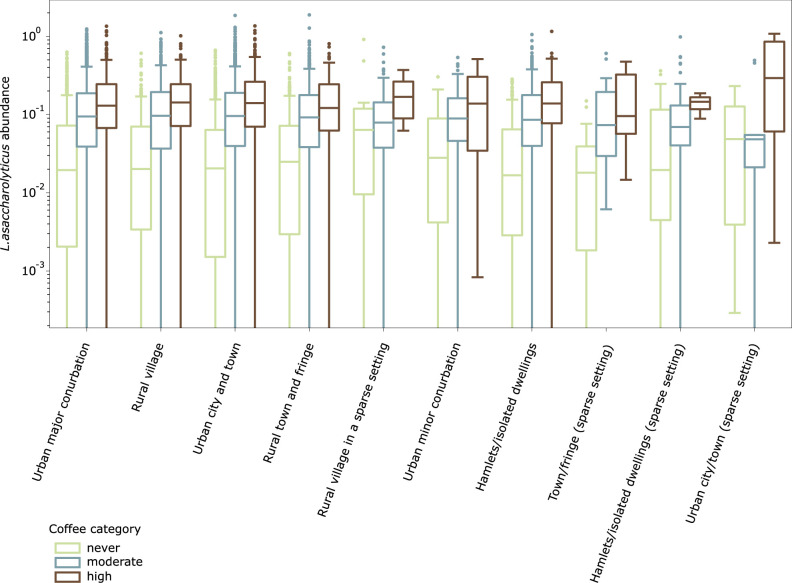
Different styles of urban and rural surroundings do not impact *L. asaccharolyticus*-coffee association*.* *L. asaccharolyticus* relative abundance in never, moderate, and high coffee-drinkers and ten different rural/urban contexts that were available in the PREDICT3 UK22A cohort (n = 11,547). Boxes represent the median and interquartile range (IQR) of the distributions; top and bottom whiskers mark the point at 1.5 IQR.

Among the top coffee-associated SGBs, *L.* *asaccharolyticus* showed the highest and the most uniform prevalence across all the cohorts (93.5%; Fig. 2c). In the ‘never’ group from the USA, its prevalence was uniformly high (average prevalence of 87.8 ± 2%) across nine different regions (samples from PREDICT2 and PREDICT3 US22A, *n* = 9,210). Over and above this, however, it was uniformly increased in all regions when considering coffee consumption; it increased from 87.8% to 95.6% in moderate drinkers and from 95.6% to 97.7% in high drinkers (Fig. 2d and Supplementary Table 11). Degree of urbanization (rural versus urban living context) was not associated with *L.* *asaccharolyticus* in the microbiome (Extended Data Fig. 6 and Supplementary Table 12). Overall, the median abundance of *L.* *asaccharolyticus* ranged from 4.5- to 8-fold higher in the high compared with the never group (in the PREDICT3 US22A and MBS–MLVS cohorts), and 3.4- to 6.4-fold higher in the moderate versus the never group (in the PREDICT2 and MBS–MLVS cohorts; Supplementary Table 13). By contrast, the highest median fold change between moderate and high drinkers was only 1.4 and did not reach statistical significance in three out of five cohorts (Fig. 3a and Supplementary Table 14).

To test whether these associations are at least partially due to a direct effect of coffee on *L.* *asaccharolyticus*, we performed in vitro experiments by supplementing coffee on *L.* *asaccharolyticus* cultures. To this end, the type strain *L.* *asaccharolyticus* DSM106493 was separately cultured with two selected common coffee preparations, that is, moka brewed and instant coffee (Methods). For both coffee preparations, we also tested the commercially available decaffeinated variants. The growth of *L.* *asaccharolyticus* was stimulated on agar plates supplemented with coffee at concentrations of 5 and 10 g l^−1^, regardless of coffee preparation (moka versus instant) and caffeine presence (one-sample *t*-test *P* = 0.02 and 0.01 for 5 and 10 g, respectively; Fig. 3b). We further tested the growth of *L.* *asaccharolyticus* associated with coffee supplementation in liquid media as assessed by optical density measurements. Despite the inherently low growth levels of *L.* *asaccharolyticus* (OD_650_ range of 0.0138–0.217), this experiment confirmed the stimulatory effect of coffee (average increase of 350% (3.5 median fold change), Dunnet’s *q* < 0.01 in ten out of 16 preparations; Fig. 3c and Supplementary Tables 15 and 16). As comparative controls, we applied the same experimental conditions to two isolates that we obtained from faecal samples of healthy donors, namely *Escherichia coli* (SGB10068) and *Bacteroides fragilis* (SGB1855) (Methods), representing both facultative and obligate anaerobes of the gut microbiome but unrelated with coffee intake in our study participants (meta-analysis *ρ* = −0.02 for *E.* *coli* and 0.01 for *B.* *fragilis*). While the *B.* *fragilis* isolate showed a slight significant (but much weaker, 8% on average) growth at 1 and 2 g l^−1^, no-clear trend was detected at 5  g l^−1^ in any of the species. A greater growth decrease was instead observed in both species at 10 g l^−1^ (−42% and −30%), suggesting an inhibitory action at higher concentrations (Fig. 3d,e and Supplementary Table 16). These results suggest that the increased abundance of *L.* *asaccharolyticus* in the gut of coffee drinkers can be due to direct fitness stimulatory effects of coffee on the bacterium.

### *L. asaccharolyticus* is ubiquitous in Western populations

**Fig. 4:**
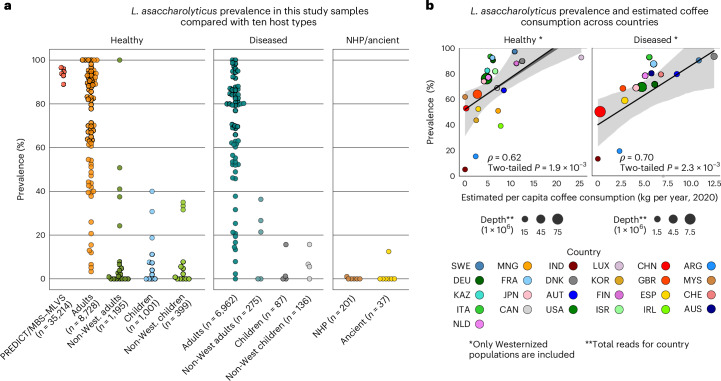
*L.* *asaccharolyticus* is ubiquitous in modern, Westernized, adult populations and almost absent elsewhere. **a**, The prevalence of *L.* *asaccharolyticus* in 11 different types of host (219 subpopulations, *N* = 54,198) including children and adults; healthy and diseased participants; from Westernized and non-Westernized communities; non-human primates and ancient samples, compared with the ZOE PREDICT and MBS–MLVS cohorts. Human, modern samples and participant records were obtained from a development version of curatedMetagenomicData^35^ (Supplementary Table 17). **b**, The per capita coffee consumption (kg per year, estimated by https://worldpopulationreview.com) for 25 countries (AUT, Austria; CHE, Switzerland; DEU, Germany; DNK, Denmark; ESP, Spain; FIN, Finland; FRA, France, GBR, UK; IRL, Ireland; ITA, Italy; LUX, Luxembourg; NLD, Netherlands; SWE, Sweden; CHN, China; IND, India; ISR, Israel; JPN, Japan; KAZ, Kazakhstan; KOR, Korea; MNG, Mongolia; MYS, Malaysia; ARG, Argentina; CAN, Canada; AUS, Australia) correlates with the prevalence of *L.* *asaccharolyticus* in healthy and diseased populations. The shaded areas around the regression line represent the 95% confidence interval estimated by bootstrapping.

We then aimed to survey the prevalence of *L.* *asaccharolyticus* across more diverse populations, by exploiting the availability of metagenomes and curated sample metadata in curatedMetagenomicData^35^. We analysed 11 categories of hosts differing in age group, health status, lifestyle and species (*N* = 54,198) for the presence of *L.* *asaccharolyticus* in 43 countries (Methods and Supplementary Table 17). *L.* *asaccharolyticus* prevalence was above 60% in 52 out of 74 cohorts (70%; Fig. 4a), with a median prevalence of 75%, mostly representing adult populations in urbanized Western-lifestyle environments (Fig. 4a). In contrast, its prevalence in individuals belonging to rural societies with non-typically Western lifestyles (20 cohorts) was much lower (median prevalence 2.4%), and *L.* *asaccharolyticus* was also only rarely found in newborns and children. Considering the available metagenomic data from non-human primates (201 samples; Methods), *L.* *asaccharolyticus* was detected in only one sample. All these lines of evidence point at a dependency of the population-level prevalence of *L.* *asaccharolyticus* with the broad availability of coffee in the diet in the population, a hypothesis further strengthened by the detection of this species in only two of the 37 samples from ancient populations we had access to (Fig. 4a and Supplementary Table 17).

**Extended Data Fig. 7:**
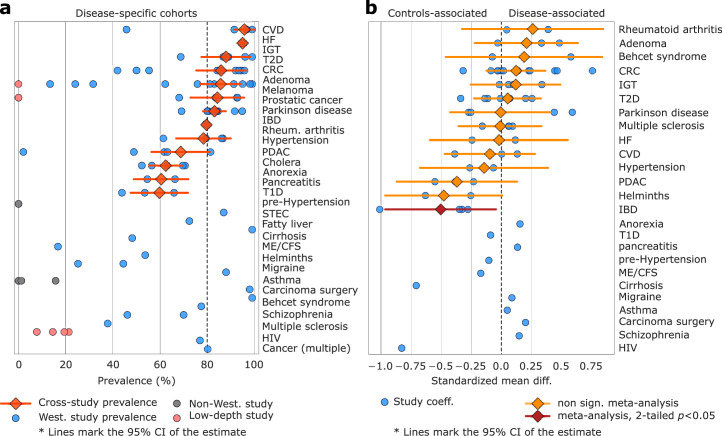
*L. asaccharolyticus* in relation with sequencing depth and in 35 disease types. **a)** *L. asaccharolyticus* is highly prevalent in patients from 35 diseases from 89 combinations of study + country + disease, when prevalence across same-disease datasets was computed via random-effect meta-analysis. **b)** Standardized mean difference of arcsin-square rooted abundances of *L. asaccharolyticus* in public case-control settings from 25 disease-types shows no strong association between *L. asaccharolyticus* and diseases. Effect-sizes of the same-disease datasets were computed via random effect meta-analysis. Total sample sizes are available in Supplementary Table 18,19.

Analysing microbiome samples from previous studies of various diseases (Methods), *L.* *asaccharolyticus* prevalence was comparably high in 7,004 samples from non-healthy adults and children from Westernized populations as well as 411 from non-Westernized populations (Fig. 4a and Supplementary Table 17). Prevalences ≥80% were found in all but one cancer type tested and in cardiometabolic diseases, and also no differences were found when meta-analysing studies with case–control metagenomic information across 25 diseases (7,154 controls and 5,670 cases; Extended Data Fig. 7a,b and Supplementary Table 18 and 19).

### *L. asaccharolyticus* correlates with worldwide coffee intake

To investigate whether different average coffee consumption rates are the potential drivers of the variable prevalence of *L.* *asaccharolyticus* in the Westernized populations (95% confidence interval, 40–97%), we took advantage of the available data to directly correlate estimated annual coffee consumption with *L.* *asaccharolyticus* prevalence (Methods). Country-level coffee consumption was strongly correlated with *L.* *asaccharolyticus* prevalence (*ρ* = 0.62 and 0.70, *P* = 1.9 × 10^−3^ and 2.3 × 10^−3^ in healthy and diseased cohorts, respectively; Fig. 4b). This finding strongly reinforces the hypothesis that not only is *L.* *asaccharolyticus* abundance in a person stimulated by their coffee intake but also the overall prevalence in a population is driven by the population-level average coffee consumption.

### Coffee plasma metabolites linked with *L. asaccharolyticus*

**Fig. 5:**
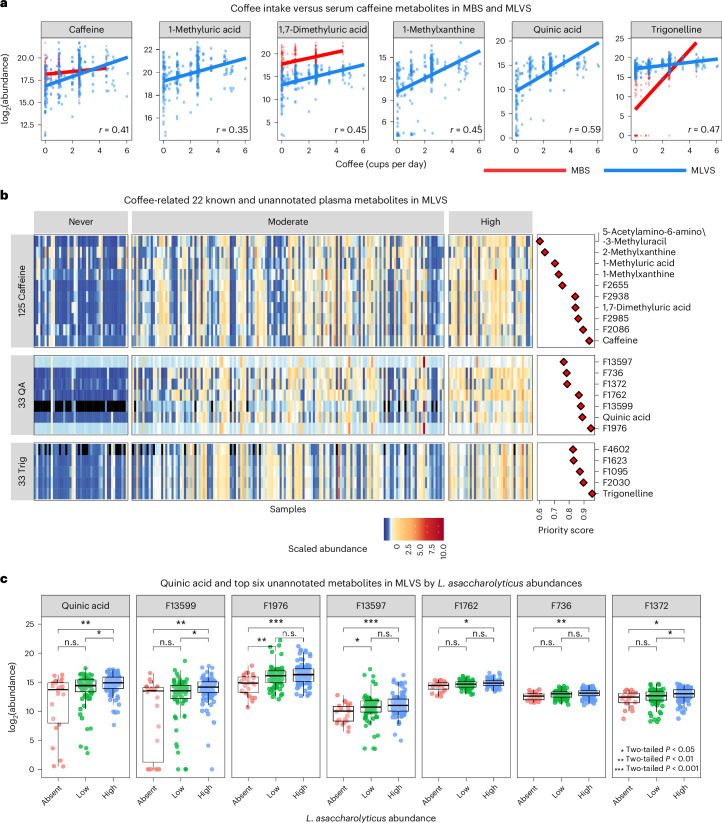
Unannotated metabolites covarying with quinic acid are associated with *L.* *asaccharolyticus.* **a**, The correlation of coffee intake versus abundances of six known coffee metabolites in plasma metabolomics samples from the MLVS (blue) and MBS (red). The highest rank correlation is reported in each plot. Three metabolites were not measured in MBS. **b**, Left, a heat map showing standardized abundances of the 14 unannotated and 8 previously annotated metabolites in the MLVS cohort (*n* = 307) with the highest MACARRoN priority score with respect to the presence of *L.* *asaccharolyticus*. QA, quinic acid; Trig, trigonelline. Right, MACARRoN priority scores. Samples are reported by coffee intake category. **c**, The log_2_-transformed abundances of quinic acid and the top six quinic acid-correlated unannotated metabolites according to *L.* *asaccharolyticus* relative abundance (RA) categories (absent, RA <0.01%; low, 0.1%> RA ≥0.01%; high, RA >0.1%) in 190 coffee drinkers. The boxes represent the median and IQR of the distributions, and top and bottom whiskers mark the point at 1.5 IQR.

**Extended Data Fig. 8:**
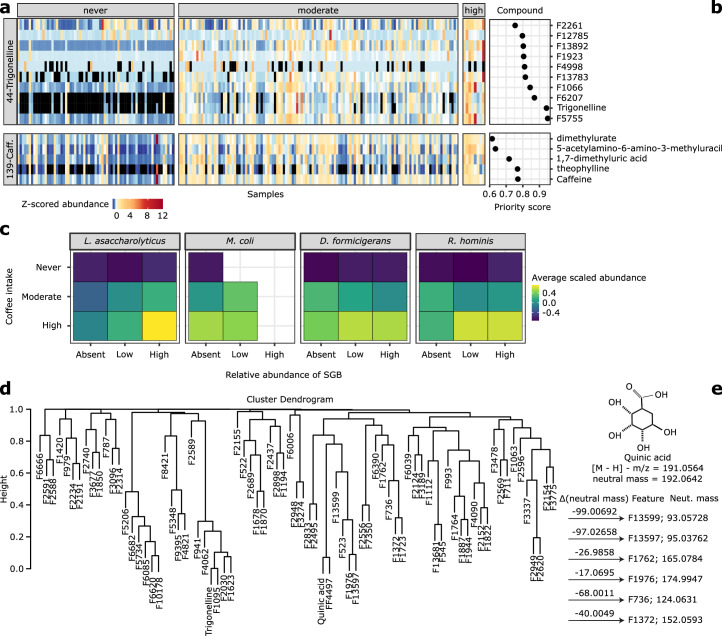
Unannotated metabolites covarying with trigonelline and caffeine in a *L.* asaccharolyticus-dependent manner*.* **a)** Heatmap showing the standardized abundance of 15 plasma metabolites in the MBS cohort (n = 213) showing the highest MACARRoN priority score for the presence of *L. asaccharolyticus*. **b)** MACARRoN priority scores for these metabolites. Samples are reported by coffee intake category. **c)** Mean scaled (z-score across metabolomes) relative abundances of the 25 metabolites in metabolomes that belong to one of the nine categories in the MLVS cohort. Enrichment of these metabolites is most prominent in *L. asaccharolyticus* and simultaneously linked to both higher coffee consumption and relative abundance (RA) of the species (Absent: RA < 0.01%; Low: 0.1% > RA ≥ 0.01%; High: RA > 0.1%). For the non coffee-associated SGBs, enrichment is seen to be linked only to higher coffee intake. **d)** Clustering dendrogram of the MACARRoN between metabolites correlations. **e)** Quinic acid structure and mass-to-charge ratio suggests that six unannotated metabolites prioritized by MACARRoN are derived by modification of quinic acid.

We next analysed 235 MLVS and 203 MBS plasma metabolomes spanning a total of ~14,000 metabolic features each (Methods). These included six metabolites in the caffeine metabolism pathway^36^, including caffeine, 1-methyluric acid, 1,7-dimethyluric acid, 1-methylxanthine, 3-methylxanthine and 5-acetylamino-6-amino-3-methyluracil, as well as quinic acid and trigonelline. We observed a significant positive correlation between these eight coffee metabolites and coffee intake in both cohorts (Fig. 5a). Next, we used MACARRoN^37^, a bioactive metabolite prioritization workflow to find modules (clusters) of covarying metabolic features associated with *L.* *asaccharolyticus* in each cohort (Methods). In the MLVS metabolomes, known coffee metabolites were prioritized to be associated with *L.* *asaccharolyticus* (Fig. 5b). Additionally, they covaried with several unannotated features in two modules: one (module 125) containing caffeine and its derivatives, while the other (module 33) containing trigonelline and quinic acid (Fig. 5b). Similarly, in the MBS cohort, both caffeine- and trigonelline-related metabolites (quinic acid was not measured in this cohort) were found to be separated into different modules (Extended Data Fig. 8a,b).

### *L. asaccharolyticus* and coffee enrich quinic acid metabolites

In addition to the known metabolites, unannotated metabolic features that strongly correlated with caffeine and quinic acid in modules 125 and 33, respectively, were also prioritized by MACARRoN as associated with *L.* *asaccharolyticus* (Supplementary Table 20). Furthermore, similar to known coffee metabolites, these unannotated prioritized features were enriched in moderate and high coffee drinkers, thus corroborating their correlation with *L.* *asaccharolyticus* (Fig. 5b). To further confirm the uniqueness of the associations between coffee-associated SGBs and coffee-associated metabolites, we repeated the aforementioned analyses and prioritized for another coffee-linked species *M.* *coli* (Fig. 2a) and two other species, *Roseburia hominis* (SGB4936) and *Dorea formicigenerans* (SGB4575), that are not coffee-associated but have the same prevalence and abundance patterns as *L.* *asaccharolyticus* in the MLVS cohort. As expected, coffee-associated metabolites were prioritized only in *M.* *coli* (Supplementary Table 21 and Extended Data Fig. 8c).

Because an interaction model identified a significant effect between coffee intake and *L.* *asaccharolyticus* only for quinic acid (interaction *P* = 3.39 × 10^−13^; Supplementary Table 22), this raised the hypothesis that the association of the microorganism with coffee may be particularly related to the biochemistry of the six unannotated compounds in module 33 that strongly correlated with quinic acid (Fig. 5b and Extended Data Fig. 8d). Testing this among coffee drinkers in the MLVS cohort (*n* = 190), individuals with higher abundance of *L.* *asaccharolyticus* showed higher abundance of quinic acid and related prioritized compounds (Fig. 5c). Upon examination of their masses, we found that they all differed from quinic acid by a small mass difference, suggesting that they are potential derivatives of quinic acid, although we could not confidently assign their identities (Extended Data Fig. 8e). One of them, F1976 (neutral mass 174.9947), which correlated with quinic acid (*ρ* = 0.43), was similar in mass to shikimic acid (monoisotopic molecular weight of 174.0528 g mol^−1^), which is synthesized from quinic acid by gut microorganisms^11^^,38^ (Extended Data Fig. 8d,e). We also observed a feature in this module that potentially represents pyrogallol (monoisotopic molecular weight of 126.03 g mol^−1^; [M + H], 127.0389; neutral mass, 126.0311; feature, F787), another gut microbial derivative of dehydroshikimic acid and quinic acid^11^, although it was not differentially abundant in participants carrying *L.* *asaccharolyticus*. Together, this analysis suggested the presence of pathways responsive to coffee and, more specifically, quinic acid in *L.* *asaccharolyticus*.

**Extended Data Fig. 9:**
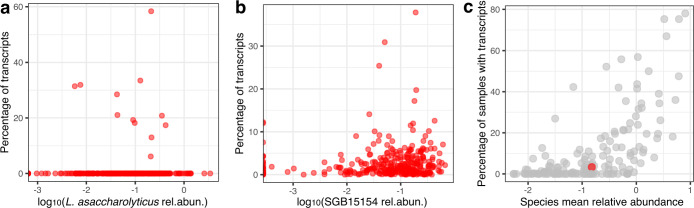
Metatranscriptomics of MLVS. **a)** *L. asaccharolyticus* transcripts are detected in only twelve samples by metatranscriptomics. **b)** SGB-level profiling reveals that a large majority of samples contain *L. asaccharolyticus* (SGB15154) transcripts however transcript coverage is low. **c)** Species abundance and transcript detection are correlated where transcripts from higher abundance species are detected in a larger number of samples compared to transcripts from lower abundance species. The red dot indicates *L. asaccharolyticus*.

Towards this, we identified *L.* *asaccharolyticus* transcripts in metatranscriptomes of 364 samples from the MLVS cohort for which corresponding species abundance data were available. Transcripts of only 1,225 total UniRef90 protein families (146 Enzyme Commission (EC) numbers) were attributed to *L.* *asaccharolyticus* and were detected in only 12 (3.29%) samples (Methods). Moreover, there was no correlation between *L.* *asaccharolyticus* abundance and number of transcripts detected per sample, and only one sample contained more than 50% (*n* = 715) of all detected *L.* *asaccharolyticus* UniRef90 families (Extended Data Fig. 9a–c). To further improve transcript detection, we carried out SGB-level functional profiling using a custom bowtie database (Methods), which increased the number of detected UniRef90s from 1,225 to 3,158 in 352 (96.7%) samples. Despite this significant improvement in the number of samples containing at least one *L.* *asaccharolyticus* transcript, only 14 (3.84%) samples contained >10% of the total transcripts and only two contained >25% transcripts. Our integrated metabolomic, metagenomic and meta-transcriptomic analysis thus allowed us to identify the pathways probably connected with coffee metabolism, although single-transcript analysis did not have enough resolution to confirm the increased activity of the single genes in such pathways, a common phenomenon for low-abundance species that are neglected by highly abundant and highly transcribing species^39^.

## Discussion

Here, we investigated the relationship between coffee intake and the gut microbiome by analysing over 22,000 metagenomes of participants across five US and UK cohorts. The gut microbiomes of coffee drinkers were clearly distinguishable from those of non-drinkers, also with an effect of the amount of coffee consumption. A set of 115 SGBs were positively associated with coffee intake (*q* < 0.001), and among these, *L.* *asaccharolyticus* showed the strongest association, with its median abundance 4.5–8-fold higher in coffee drinkers compared with non-drinkers. This link between *L.* *asaccharolyticus* and coffee consumption was confirmed when correlating estimated per capita coffee intakes and *L.* *asaccharolyticus* prevalence in 25 countries with re-analysis of many thousands of public metagenomes (Fig. 4b).

Among the 115 positively coffee-associated species, we identified some SGBs that were already highlighted by previous work including two *Faecalibacterium* and two *Alistipes* SGBs. Nonetheless, our analysis did not confirm the same patterns for other genera such as *Bacteroides*, *Porphyromonas* or *Prevotella*, which were identified by previous studies^16,22,23^. These unconfirmed associations could be cases of cohort-dependent associations. Importantly, most of the biomarkers identified in our study belonged to uncharacterized genera and families (mostly Clostridia). Out of the top 50 associations, for example, 36 were poorly characterized, and 6 were labelled as ‘Candidatus’ species. *L.* *asaccharolyticus* was, in line with this observation, only isolated in 2018 (ref. ^26^), further highlighting the necessity of expanding knowledge on uncharacterized bacteria.

The in vitro cultivation experiments demonstrated the stimulatory potential of coffee on the growth of *L.* *asaccharolyticus*, especially at concentrations that inhibited two commensal species (Fig. 3b–e). These findings lay the groundwork for future experiments aimed at further characterizing the extent of the stimulatory effect of coffee on *L.* *asaccharolyticus*, by testing varying concentrations of coffee with respect to more control microorganisms. In addition, metabolomic analysis revealed that quinic acid, trigonelline and correlated unknown metabolites are significantly enriched in coffee drinkers carrying *L.* *asaccharolyticus*. The top ten coffee-associated SGBs remained strongly correlated (*q* < 0.001) with decaffeinated coffee consumption, indicating that their underlying biochemistry is probably caffeine independent (Fig. 2b and Extended Data Fig. 4b). Chlorogenic acid, one of the main polyphenols in coffee, is metabolized extensively by gut microorganisms first to caffeic acid and quinic acid, and further to dihydroferulic acid, dihydrocaffeic acid, vanillin, benzoic acid, 3-(3′-hydroxyphenyl)propionic acid and pyrogallol, among others^11,12,21^. Several microbial species including *Bifidobacterium animalis*, *Bifidobacterium lactis*, *E.* *coli* and *Lactobacillus gasseri* have been implicated in the aforementioned biotransformations^11,38,40^. The strong association of *L.* *asaccharolyticus* with coffee independently of caffeine indicates that it may also be responding to activities within these polyphenol metabolism pathways.

In this vein, a recent study reported enrichment of *L.* *asaccharolyticus* in 51 participants following aronia berry consumption, which contains several compounds overlapping with coffee including chlorogenic acid, caffeic acid and other benzoic and cinnamic acids^41^. Another study specifically implicated *L.* *asaccharolyticus* as the mediator between coffee intake and the presence of caffeine derivatives such as 5-acetylamino-6-amino-3-methyluracil and 1,3-dimethyluric acid in human stools^24^. On the basis of our results, caffeine and its covarying derivatives were prioritized due to their association with *L.* *asaccharolyticus*, but the enrichment of the microorganism in decaffeinated coffee drinkers indicates that caffeine is unlikely to fully explain the overall chemistry. Instead, these unannotated metabolites might be derivatives of quinic acid produced by *L.* *asaccharolyticus* (Fig. 5c). For example, quinic acid is converted to pyrogallol by the gut microbiome in a multi-step process that includes dehydroshikimate and shikimic acid as intermediates^11,38,40^. We detected a metabolic feature that potentially represents pyrogallol (F787) in the quinic acid module, although the feature is not prioritized possibly due to its poor absorption into the bloodstream from the colon. A biomarker of metabolic and gut health, hippurate^42^, was also prioritized and enriched in *L.* *asaccharolyticus-*carrying participants. Hippurate is also a glycine conjugate derived from benzoic acid and quinic/shikimic acid, and synthesized by the gut microbiome metabolism of dietary compounds^43,44^.

While several metabolites are thus known to be produced by the degradation of quinic acid by gut microorganisms^11,45^, knowledge of the enzymes that catalyse these reactions is mostly lacking. A recent study proposes two routes of degradation: (1) oxidation to protocatechuic acid, and (2) reduction to hexahydrobenzoic acid. Speculations about participating enzymes can be made on the basis of individual biotransformations such as dehydroxylation and aromatization, although assigning these functions to specific genes of specific taxa is challenging. Our efforts to identify participating enzymes in *L.* *asaccharolyticus* were stymied by the lack of detectable transcripts: only ~4% of samples contained >10% of the total detected *L.* *asaccharolyticus* transcripts, which were themselves not indicative of complete transcriptome detection. This is a common phenomenon for taxa that are present in low DNA abundance^39^ (Extended Data Fig. 9a–c).

Our study provides insights into how the gut microbiome potentially mediates the chemistry—and thus health benefits—of coffee. Up to 115 SGBs responded positively to coffee intake, highlighting the impact that a single daily food item can have on the human gut microbiome. Still, the strongest link is by far that with *L.* *asaccharolyticus*, and, as such, efforts of revealing underlying mechanisms of coffee stimulation in vitro should be the natural next step. With coffee intake that has been implicated in all-cause mortality risk, future work should try to establish whether this link is potentially mediated by *L.* *asaccharolyticus*. The microbial mechanisms underlying the metabolism of coffee are a step towards mapping the role of specific foods on the gut microbiome, and similar patterns of microorganism–food interactions for other dietary elements should be sought with systematic epidemiologic and metagenomic investigations.

## Methods

### Ethical compliance

All cohorts collection procedures complied with all ethical regulations, including the Declaration of Helsinki (2013). Ethical approval of the MBS and MLVS cohorts was granted by the institutional review boards (IRBs) of the Brigham and Women’s Hospital and the Harvard T.H. Chan School of Public Health. The ZOE PREDICT cohorts were approved in the UK by the Research Ethics Committee and Integrated Research Application System (IRAS 236407), and in the USA by the Institutional Review Board: Partners Healthcare IRB 2018P002078, clinical trial NCT03479866 (ZOE PREDICT1), IRB Pro00033432, NCT03983733 (ZOE PREDICT2) and NCT04735835 (ZOE PREDICT3). Any procedure involving individuals from all cohorts was carried out after having received written informed consent.

### The ZOE PREDICT cohorts

We included in this study five cohorts from the ZOE PREDICT programme^27^. PREDICT1, which includes 1,098 participants from the UK aged 18–66 years (792 female (72%), 5.8 × 10^10^ reads in total) and was previously published^25^; PREDICT2 (*n* = 975, individuals from the USA aged 18–84 years (703 female (72%), 5.8 × 10^10^ reads sequenced in total); PREDICT3 US21 (*n* = 11,798 participants from the USA aged 18–87 years (10,270 female (87%), 4.4 × 10^11^ reads in total); PREDICT3 US22A (*n* = 8,470 participants from the USA aged 18–90 years (7,361 female (87%), 3 × 10^11^ reads); PREDICT3 UK22A (*n* = 12,353 participants from the UK aged 18–95 years (9,447 female (76%), 3.9 × 10^11^ reads in total). These cohorts are characterized by detailed monitoring of the nutritional habits of the customer, customer personal and anthropometric characteristics, and collection of stool samples. The questionnaire type adopted varied across cohorts. The European Prospective Investigation into Cancer and Nutrition FFQ (131 items in total)^46^ that captures, at compiling time, the food habits in the past year, was used in PREDICT1. A 135-item similar questionnaire (DHQ-III)^47^ was used in PREDICT2. PREDICT3 UK22A and US22A were surveyed by a questionnaire of 264 food items (Linenberg, I. et al., manuscript in preparation). FFQ-derived food item consumptions were grouped into categories by a ‘food tree’ built as a nutrient estimation-based database structured in a level one (nine food groups), a level two (52 groups) and a level three (195 possible food groups). The level three groups were fine grained enough to permit the analysis of total coffee consumption, dairy cream, sugars and honey, and milk independently, while to analyse the intake of caffeinated and decaffeinated coffee, we went back to the original value from the FFQ information. The solidity of a similar approach for the collection of habitual diet information with FFQ-based information from cohort participants is described elsewhere (Leeming, E. et al., manuscript in preparation). The PREDICT3 US21 had dietary information based on short-term logged dietary, thus was the sole cohort not based on (previously established) long-term FFQ-based information and as such was not included in the analysis of food items. It was only analysed together with other ZOE PREDICT cohorts in the context of the microbial epidemiology of *L.* *asaccharolyticus*. The total size of the four cohorts analysed for their dietary preferences was decreased to the individuals with available dietary information. As such, PREDICT1 and PREDICT2 sizes were downsized to 928 and 844, respectively. Sample sizes of the four PREDICT cohorts with available dietary information were further decreased by removing individuals resulting in a coffee consumption >99th percentile of the cohort. No other statistical method was used to predetermine sample size. The final numbers of samples considered were 914 individuals for PREDICT1, 835 for PREDICT2 cohort, 8,376 for PREDICT3 US22A and 12,222 for PREDICT3 UK22A. The coffee estimated intake of the PREDICT1 cohort was used as a reference for coffee intake participant categories as done in ref. ^25^. In brief, participants with an intake of coffee below 20 g per day (0.7 cups per day or 3 cups per week), were assigned to the ‘never’ class. Participants with an intake of coffee superior to 20 g per day and inferior to 600 g per day (3 cups per day) were assigned to the ‘moderate’ category. Participants with a coffee intake greater than 600 g per day were assigned to the ‘high’ category. The two thresholds used for PREDICT1 corresponded to the 24.9th and the 88.95th percentile of the overall coffee intake distribution. These percentiles were applied on the other cohorts independently. The resulting numbers are resumed in Supplementary Table 2.

### The MBS and MLVS cohorts

The MLVS^29^ is a substudy of the main Health Professionals Follow-up Study (HPFS)^48^ and consisted of 700 men aged 52–81 years who were free of coronary heart diseases, stroke, cancer (except squamous or basal cell skin cancer) and major neurological diseases. From 2012 to 2013, a total of 307 men in the MLVS provided up to two pairs of self-collected stool samples from consecutive bowel movements. Each pair of samples were collected 24–72 h apart and the two pairs were collected approximately 6 months apart^29^. Two blood samples were drawn, 6 months apart, to coincide with the timing of the faecal sample collection. The MBS is a subcohort nested in the Nurses’ Health Study II (NHSII) (https://nurseshealthstudy.org), which adopted the same protocol for stool sample collection as in the MLVS^28^. The NHSII is an ongoing prospective cohort study of 116,429 US female nurses aged 25–42 years at enrolment in 1989. During 2013–2014, 233 women from the MBS were mailed stool and blood sample collection kits and 209 women returned usable stool samples. In a combination of MLVS and MBS, we analysed 520 individual metagenomes, 464 metatranscriptomes from 64 participants and 438 metabolomes. At the time of stool sample collection, a 116-item FFQ was administered to the MLVS and MBS participants to collect information on diet including coffee intake in the previous year. On the FFQs, participants were asked about the frequency, on average, of coffee consumption during the previous year, with one cup as the standard portion size. The consumptions of caffeinated coffee and decaffeinated coffee were assessed separately. Nine possible responses ranged from never or <1 time/month to ≥6 times per day. The nutrient intake was computed by first multiplying the frequency of consumption for each food by its nutrient content and then summing up nutrient contributions across all food items. Both caffeinated and decaffeinated coffee intake were assessed in the FFQs and the sum of the two was calculated as the consumption of total coffee. The validity and reproducibility of the FFQs have been described in detail elsewhere^49^. No statistical method was used to further predetermine sample size. The study was approved by the human participants committees at Brigham and Women’s Hospital and Harvard T.H. Chan School of Public Health. All participants provided written informed consent.

### Reads pre-processing, taxonomic and functional profiling

Metagenomic sequences from the ZOE PREDICT were pre-processed with the pipeline available at https://github.com/SegataLab/preprocessing; the MBS and the MLVS cohorts were processed with KneaData 0.3 (http://huttenhower.sph.harvard.edu/kneaddata). In both pipelines, reads were aligned to the human genome hg19 and to the PhiX174 genome to remove contaminants and the Illumina spike. In the PREDICT cohorts, non-aligning reads were extracted with samtools^50,51^ and quality-screened with Trim Galore (--stringency 5 --length 75 --quality 20 --max_n 2 --trim-n)^52^ to remove reads <75 bp and with Phred quality <20 as well as with more than two ambiguous nucleotides. High-quality reads of all cohorts were taxonomically profiled by the SGB system^53^ using the January 2021 release of MetaPhlAn 4 (ref. ^30^). Metatranscriptomics sequences from MLVS were also pre-processed using KneadData 0.3. Transcriptome profiling of the MLVS cohort was performed once using the algorithm of HUMAnN 3.6 (ref. ^54^). To account for uncharacterized SGBs as well, we built SGB-level pangenomes collecting all the UniRef90 gene families present in a minimum of one genome (metagenome-assembled genome (MAG) or isolate) of the SGB. This was indexed and passed as a nucleotide database (--nucleotide-database). Since as few as 1,225 UniRef90 gene families were attributed to *L.* *asaccharolyticus*, we performed an additional run of HUMAnN 3.6 on a custom database considering only the pangenomes of the top ten coffee-associated SGBs to reduce mapping competition and highlighting up to 3,158 gene families that were attributed to the *L.* *asaccharolyticus* pangenome (--bypass-nucleotide-index --bypass-translated-search).

### Taxonomic assignment of *L. asaccharolyticus*

Large-scale clustering of MAGs using an average nucleotide identity threshold of 95% (ref. ^53^) defined the SGB cluster ID 15154 that comprises 207 MAGs and three reference genomes from the National Center for Biotechnology Information. These three references are GCA_001244495 and GCA_902375485 (both assigned to the *Clostridium phoceensis* taxonomic label, tax id 1650661)^34^ and GCA_003112755 (assigned to the *L.* *asaccharolyticus* taxonomic label, tax ID 2108523)^26,33^. The *L.* *asaccharolyticus* taxonomic label comprises the isolate genome registered and publicly available from the German Collection of Microorganisms and Cell Cultures GmbH (DSMZ) repository (type strain 106493). On the contrary, *C.* *phoceensis* does not have any deposited isolate available from the DSMZ nor American Type Culture Collection repositories and it is not validly published as a species (https://lpsn.dsmz.de/species/clostridium-phoceensis). We then concluded that the most reliable taxonomic label assigned to these three reference genomes is the *L.* *asaccharolyticus*. However, due to the majority voting rule, currently the SGB ID 15154 when profiled by MetaPhlAn 4 (ref. ^30^) reports the taxonomic label *C.* *phoceensis*. This will be corrected from future database releases of bioBakery 4.

### Bacterial cultivation

The in vitro experiments to test the effect of coffee on *L.* *asaccharolyticus* were performed with the publicly deposited *L.* *asaccharolyticus* strain and two bacterial strains from the *E.* *coli* and *B.* *fragilis* species isolated from stool samples and used as controls. *L.* *asaccharolyticus* DSM106493 strain was obtained from the Leibniz Institute DSMZ Culture Collection. The *E.* *coli* and *B.* *fragilis* strains were instead isolated from stool samples of two healthy adults at University of Trento (protocol no. 2021-007 by the Ethical Committee of the University of Trento) as described in ref. ^55^. For all coffee supplementation tests performed in this study, each strain was pre-cultured at 37 °C in Brain Hearth Infusion (BHI) broth supplemented with 1 μl ml^−1^ vitamin K1 (Sigma-Aldrich, 95271) and 5 mg l^−1^ hemin (Sigma-Aldrich, 51280) under anaerobic conditions (75% N_2_, 20% CO_2_ and 5% H_2_) without shaking.

### Testing the effect of coffee supplementation on bacterial monocultures in vitro

We tested the effect of coffee on *L.* *asaccharolyticus* growth by cultivation onto BHI agar plates supplemented with different coffee preparations, including brewed coffee, brewed decaffeinated coffee, instant coffee and instant decaffeinated coffee. We tested coffee supplementation concentrations at 0, 5, 10 and 20 g l^−1^. Coffee solutions were prepared as follows. Instant coffee powder (Nescafé Gold soluble) and instant decaffeinated coffee powder (Nescafé Gold decaf soluble) were weighed and dissolved in distilled H_2_O. Normal moka brewed coffee (Illy tostato classico, illycaffè S.p.A., 7189ME) and decaffeinated moka brewed coffee (Illy decaffeinato, illycaffè S.p.A., 7187ME) solutions were prepared using a moka pot. Each type of coffee powder was weighed and placed on the filter basket, and the volume of water in the bottom chamber was measured before brewing. All coffee preparations were individually sterilized by autoclaving and were subsequently added to the BHI agar medium anaerobically before pouring the plates. *L.* *asaccharolyticus* liquid culture was grown to exponential phase (OD_650_of 0.1–0.15) and serially diluted to plate at a final concentration of 1 × 10^−4^ or 1 × 10^−5^ colony-forming units (c.f.u.) per ml and 100 μl of each culture dilution was plated onto different coffee-supplemented BHI agar plates. The plates were incubated anaerobically at 37 °C for 5 days, after which visible colonies were counted. The c.f.u. per ml were reported for 10^−4^ dilution in which ~10–300 single colonies were visible. Statistical significance was determined via one-sample *t*-test comparing coffee-treated plates at a given concentration with the corresponding control value as zero value.

The effect of supplementation of the four coffee preparations on bacterial liquid growth was separately assessed for *L.* *asaccharolyticus*, *B.* *fragilis* and *E.* *coli*. Single bacterial pre-cultures were inoculated to reach an initial OD_650_ of 0.001 into BHI broth supplemented with the four single coffee preparations at concentrations of 0, 1, 2, 5 and 10 g l^−1^, anaerobically. Tests were performed using 2 ml 96-well plates, with five technical replicates per tested bacterial species and per coffee concentration. Bacterial growth in the presence of coffee was assessed by monitoring changes in optical density (OD_650_) using a microplate spectrometer (Infinite M200 Pro, Tecan) at 48 h and is reported as the percentage compared with growth in the non-supplemented medium control (0 g l^−1^ coffee ctrl, 100%). For *E.* *coli*, the conditions of supplementation with instant coffee 5 g l^−1^, instant decaffeinated coffee 5 g l^−1^ and brewed decaffeinated coffee 1 g l^−1^ were excluded due to contamination assessed by 16S rRNA gene amplicon sequencing. Median growth compared with controls was assessed via Kruskal–Wallis followed by a post hoc Dunnett test with Holm correction at a statistical significance threshold of *q* < 0.01.

### Metabolomic analysis

Intensities of 13,829 chemical features were obtained from 235 plasma metabolomes in the MLVS cohort using three liquid chromatography–mass spectrometry methods: (1) C8 positive for polar and non-polar lipids, (2) hydrophilic interaction liquid chromatography (HILIC) negative and (3) HILIC positive for polar metabolites. Similarly, 14,095 features were quantified from 203 plasma metabolomes in the MBS cohort using HILIC-positive, C18-negative and C8-positive liquid chromatography–mass spectrometry methods. Owing to the lack of HILIC-negative columns in the MBS metabolomic analysis, metabolites measured by that column, for example, 1-methyluric acid, 1-methylxanthine and quinic acid, were analysed in MLVS only. Chemical identities of 393 and 391 features in the MLVS and MBS cohorts, respectively, could be determined by matching recorded retention time and *m*/*z* values to an internal database of >600 compounds. For both metabolomics datasets, raw data were processed as described earlier^56^ and median-normalized intensities were used for all downstream analyses. To assess the strength of associations between coffee-associated compounds and coffee-associated (*L.* *asaccharolyticus*, *M.* *coli* and Clostridia 12CBH8 SGB7259) and non-associated SGBs (*D.* *formicigenerans* and *R.* *hominis*) in the MLVS cohort, we used MACARRoN^37^, a workflow that prioritizes compounds based on their predicted bioactivity in a phenotype (or condition) of interest. Metabolomes were assigned ‘phenotypes’ based on the relative abundance of the SGB in corresponding metagenomes: ‘carriers of SGB’ if relative abundance >0.01% and ‘non-carriers of SGB’ otherwise. This threshold yielded 194, 11, 7, 200 and 190 ‘carrier’ metabolomes for *L.* *asaccharolyticus*, *M.* *coli*, Clostridia 12CBH8 SGB7259, *D.* *formicigenerans* and *R.* *hominis*, respectively. To boost the number of ‘carrier’ metabolomes for *M.* *coli* and Clostridia 12CBH8 SGB7259, we relaxed the relative abundance threshold to 0.005%; this increased the numbers to 21 and 9, respectively. Due to the low number of carrier metabolomes, MACARRoN could not be applied to Clostridia 12CBH8 SGB7259. For all other SGBs, the priority or importance of each compound was inferred using a variety of indicators of bioactivity including covariance with well-studied metabolites, that is, standards, abundance with respect to a covarying standard (abundance versus anchor), prevalence and differential abundance in the studied phenotype/condition. The differential abundance of features in metabolomes corresponding to SGB was estimated with per feature linear models (feature ~ SGB carriage + age + BMI). Unannotated features with priority score >0.75 and annotated features with priority score >75th percentile of priority scores were considered to be strongly associated with the presence of the SGB. For *L.* *asaccharolyticus* alone, the prioritization was performed using the 203 MBS metabolomes as well. The interaction model was run setting *L.* *asaccharolyticus* and coffee intake as predictors and the metabolite abundances as responses.

### Public metagenomic data on different lifestyles

Via a developmental version of the curatedMetagenomicData^35^ resource, we accessed manually curated metadata for over 29,000 metagenomic samples and we queried this resource for stool-derived metagenomes from ten categories of host differing by age group, health status and lifestyle. In particular, we queried for stool metagenomes from stool samples from adult participants that were not diagnosed with a specific pathology and that were assigned to a Westernized lifestyle. In addition, only cohorts with a minimum of ten individuals and only the first timepoint was kept if multiple records were available per individual. In total we retrieved 8,728 microbiome profiles from healthy adults (74 datasets). With a similar query, we retrieved 1,001 samples (12 datasets) from newborns and children up to 12 years old not diagnosed for a specific disease and assigned to a Westernized lifestyle. Similarly, 20 datasets comprised adult individuals from non-Westernized communities (*n* = 1,195), meaning having limited direct or indirect antibiotics exposure, minimal or none heavily processed food availability, closeness to animals and do not subtend any geographical pattern, and 399 children from the same lifestyle (13 datasets). A similar definition of lifestyle has now been widely applied^53,57–63^ (Supplementary Table 17). We similarly queried this resource for individuals suffering from specific diseases. We retrieved 6,921 samples from Westernized adults (89 cohorts, considering combinations of dataset + country + disease), which were representatives of 29 diseases, and 279 samples from adults following non-Westernized lifestyles (four cohorts and two diseases). In these two sets, two populations (cohort + country + disease) totalled only four samples each, and therefore analysis in Fig. 4a and Extended Data Fig. 6b are based on 6,917 and 275 samples, respectively. We queried for stool metagenomes from both lifestyles from children up to 12 years of age (87 and 136 samples, five and four cohorts, seven diseases; Supplementary Tables 17 and 18). We then queried cohorts from case–control studies involving a minimum of five cases and five controls from adult individuals, reaching a total of 66 studies comprising 25 diseases (in total, 7,154 controls and 5,670 cases). Fourteen diseases were present with multiple datasets while eleven diseases were represented by a single study (Supplementary Table 19). We finally collected 201 samples from 22 species of non-human primates (6 studies) and 37 ancient samples derived from 7 different archaeological sites (Supplementary Table 17). In total, 18,984 microbiome samples (43 countries) were taxonomically profiled using the January 2021 release of MetaPhlAn 4.

### Meta-analytical approaches on coffee and SGB relative abundance

Spearman’s correlations were computed between MetaPhlAn 4 SGB relative abundances and total, decaffeinated-only and caffeinated-only coffee intake. Correlations were computed in each cohort independently and then summarized via an inverse-variance average meta-analysis (script available at https://github.com/SegataLab/inverse_var_weight). Weights in the meta-analysis were adjusted using the DerSimonian and Laird Tau approach^64^, which is particularly suitable for analyses with large differences in the studies’ sample sizes. Besides raw Spearman’s correlations, we also performed a meta-analysis of partial Spearman’s correlations. Partial correlations were adjusted by sex, age and BMI in all analyses. Analyses on decaffeinated-only coffee intake were performed excluding individuals not drinking only caffeinated coffee and adjusting by caffeinated coffee, and vice-versa for the analysis on caffeinated-only coffee (which was also adjusted for decaffeinated coffee). Sex was handled as a categorical variable with one degree of freedom. To compute partial correlations we used the parcor function from the pingouin Python library (version 0.5.4, Spearman), which relies on the computation of the variable variance–covariance matrix method, which is summarized in refs. ^65,66^. For each analysis independently (single and pooled), we applied the function fdrcorrection from the statsmodels Python library for Benjamini–Yekutieli false discovery rate. Additionally, we used power analysis (pingouin, version 0.5.4, power_corr) on the 130 SGBs identified by meta-analysis at a *q* < 0.001 and *ρ* ≥ 0.05, to evaluate the necessary sample size that would have been needed to reach three different levels of significance (*q* < 0.001, *q* < 1 × 10^−5^ and *q* < 1 × 10^−10^, not shown).

### Meta-analytical approaches to understand *L. asaccharolyticus* epidemiology

Combinations on study + country + disease (89 combinations, 35 diseases) were used to evaluate the prevalence of *L.* *asaccharolyticus* in specific diseases. When multiple combinations of study + country were present for a single disease, we evaluated the average prevalence in the disease by meta-analysis (with binomial distribution for the presence of the *L.* *asaccharolyticus*), using the same script as above and Paule Mandel heterogeneity^67^. To contrast same-study and same-country case and control sample abundances of *L.* *asaccharolyticus* (66 combinations, 25 diseases) we applied standardized mean difference (with Hedge adjustment for small sample bias and after transforming relative abundance with the arcsin square root) to all combination with at least ten case and ten control samples (7,154 controls and 5,670 cases). Then, we applied meta-analysis to those diseases that were present in multiple combinations (*n* = 14) using the above script with Paule Mandel heterogeneity. The per-country annual coffee intake from https://worldpopulationreview.com was correlated with the average prevalence per-country *L.* *asaccharolyticus* prevalence via Spearman’s *ρ*. The average prevalence was computed with a meta-analysis as in the first case. This analysis was performed independently for the sets of 8,724 healthy, Westernized adults, and 6,921 unhealthy, Westernized individuals.

### *L. asaccharolyticus* co-abundance and co-exclusion correlations with other SGBs

Between-SGB correlations were computed on all SGBs found in a minimum of two cohorts considering the same sample set used for the partial correlation meta-analysis (*n* = 22,867). SGBs below 10% prevalence were excluded and a standard log-ratio transformation (centred log-ratio following a zero-imputation strategy with a multiplicative replacement method, Python scikit-bio library, version 0.6.0) was applied to each sample followed by between-SGBs rank correlation.

### Machine learning approaches

We assessed the ability of the microbiome to predict distinct food items in each PREDICT cohort with dietary information separately (PREDICT1, PREDICT2, PREDICT3 US22A and PREDICT3 UK22A, total *n* = 23,115) using a custom framework with the same parameters as in ref. ^25^. Briefly, we used the random forest classification and regression algorithms with an 80/20 training and testing set split, repeated 100 times. For the classification task, food frequencies were divided into the first and last quartiles, used as the two classes to predict. Both regression and classification algorithms were trained on SGB-level features only, as estimated by MetaPhlAn 4. Classification was evaluated using the median AUC, while regression used the median Spearman’s correlation between real and predicted values^32^. We then used metAML (version 1.1)^31^ to assess the link with the microbiome between combinations of the following coffee categories: ‘never’, ‘moderate’ and ‘high’. This set of experiments was performed on the four ZOE PREDICT aforementioned cohorts and the MBS + MLVS cohorts. The ZOE PREDICT cohorts were first subset to the individuals ≤99th percentile of coffee intake to remove outliers that suggested an unreasonable amount of coffee (total *n* = 22,867). For the metAML random forest parameters, we used 1,000 estimator trees, 10% of the total number of features sampled in each tree, a minimum of 10 samples for each leaf and Shannon entropy as impurity criterion, as previously used^25,68–70^. Input features were SGB-level only relative abundances as estimated by MetaPhlAn 4. We performed three different validation assessments, the purpose of which was to exploit at their maximum the available data and that allowed to test the algorithms iteratively on all the cohorts to avoid any potential favourable bias for (1) a tenfold, ten-times cross-validation for each cohort; (2) LODO, in which each cohort is used as the testing set while training on all the others, iterated for each cohort and (3) cross-LODO, in which a tenfold, ten-times cross-validation is performed on each cohort including the left-out cohorts in the training sets. This to maximize the heterogeneity of the training set, to overcome cohort-specific differences especially when very large sample sizes are present, using the program at https://github.com/SegataLab/metaml/classification_thomas_manghi.py.

### Reporting summary

Further information on research design is available in the Nature Portfolio Reporting Summary linked to this article.

## Supplementary information

Supplementary Information Supplementary Figs 1–9 and Supplementary Tables 1–22.

Reporting Summary

Supplementary Tables 1–22. Supplementary Tables 1–22 with index.

## Data Availability

Raw metagenomic samples are provided for all participants of the ZOE PREDICT Studies. Specifically, PREDICT1 is made available as reported previously^25^ (under accession PRJEB39223) whereas the PREDICT2 and PREDICT3 US21, US22A, and UK22A cohorts are deposited in the European Bioinformatics Institute (EBI) under accession numbers PRJEB75460, PRJEB75462, PRJEB75463 and PRJEB75464. Sex, age, BMI, country and the quantitative taxonomic profiles are available for each sample within the curatedMetagenomicData package^35^. ZOE is the owner of the pseudonymized data and metadata of the PREDICT2 and PREDICT3 studies and researchers interested in follow-up studies requiring additional specific metadata information should fill out a research request proposal at https://zoe.com/our-science/collaborate that will be evaluated by a subpanel of the ZOE scientific advisory board for their priority, relevance and in compliance with privacy and data protection regulations. Raw metagenomics and metatranscriptomics reads for the MLVS cohort are deposited at the National Center for Biotechnology Information (NCBI) under accession PRJNA354235. Owing to participant confidentiality and privacy concerns, data cannot be shared publicly and requests to access NHS/NHSII/HPFS data must be submitted in writing. According to standard controlled access procedures, applications to use NHS/NHSII/HPFS resources will be reviewed by our External Collaborations Committee to verify that the proposed use maintains the protection of the privacy of participants and the confidentiality of the data. Investigators wishing to use NHS/NHSII/HPFS data are asked to submit a brief description of the proposed project (go to https://www.nurseshealthstudy.org/researchers (email: nhsaccess@channing.harvard.edu) and https://sites.sph.harvard.edu/hpfs/for-collaborators/ for details). Further information including the procedures to obtain and access data from the Nurses’ Health Studies and Health Professionals Follow-up Study is described at https://www.nurseshealthstudy.org/researchers (email: nhsaccess@channing.harvard.edu) and https://sites.sph.harvard.edu/hpfs/for-collaborators/.

## Code availability

The bioBakery suite of tools was used to derive a great part of the analyses of this study. MetaPhlAn 4.1 was used to obtain the taxonomic abundances in microbiome samples and is available via GitHub at https://github.com/biobakery/MetaPhlAn/wiki/MetaPhlAn-4. Patterns of microbial abundances differentiating coffee consumer type were carried out using metAML version 1.1, available via GitHub at https://github.com/SegataLab/metaml/wiki using the program classification_thomas_manghi.py. Meta-analysis procedures via inverse variance weighting were performed using a Python code available via GitHub at https://github.com/SegataLab/inverse_var_weight. The MACARRoN software is described at https://github.com/biobakery/Macarron. The curatedMetagenomicData 3 package is available at https://www.bioconductor.org/packages/release/data/experiment/html/curatedMetagenomicData.html.
