# Supplementary material for: Coffee consumption is associated with intestinal Lawsonibacter asaccharolyticus abundance and prevalence across multiple cohorts
Source: Nat Microbiol. 2024 Nov 18;9(12):3120–34. doi: 10.1038/s41564-024-01858-9 (PMC11602726; doi:10.1038/s41564-024-01858-9)
Supplement: Supplementary file 1 — Supplementary Figs 1–9 and Supplementary Tables 1–22. [file 41564_2024_1858_MOESM1_ESM.pdf]

nature microbiology

Supplementary information https://doi.org/10.1038/s41564-024-01858-9

Coffee consumption is associated with intestinal *Lawsonibacter asaccharolyticus* abundance and prevalence across multiple cohorts

In the format provided by the authors and unedited

Supplementary Tables Legends

Table S1: Coffee consumption mg/day in the ZOE PREDICT1 cohort
Table S2: Cohorts summary of coffee intake of the ZOE PREDICT and MBS/MLVS cohorts
Table S3: Machine learning results summary from cross-validations, LODO, and cross-LODO
Table S4: Analyses of Spearman's correlations per cohort
Table S5: Meta-analysis of Spearman's correlations
Table S6: Analyses of partial Spearman's correlations adjusted by sex, age, and BMI
Table S7: Meta-analysis of partial Spearman's correlations
Table S8: Meta-analysis of Spearman's correlations (caffeinated coffee only)
Table S9: Meta-analysis of Spearman's correlations (decaffeinated coffee only)
Table S10: L. asaccharolyticus co-abundance and co-exclusion correlations with other SGBs
Table S11: Prevalence of L. asaccharolyticus across US regions
Table S12: Median abundance of L. asaccharolyticus in UK rural/urban areas
Table S13: Median abundance of L. asaccharolyticus across ZOE PREDICT, MBS, & MLVS cohorts
Table S14: post-hoc Dunn-tests among medians of L. asaccharolyticus in different coffee categories
Table S15: Growth-rates of L. asaccharolyticus, B. fragilis, and E. coli in solid and liquid media
Table S16: Dunnett tests and fold-changes of L. asaccharolyticus, B. fragilis, and E. coli growths
Table S17: Epidemiology of L. asaccharolyticus in 54,198 metagenomic samples from ten host types
Table S18: Epidemiology of L. asaccharoliticus in 7,200 from 31 disease types
Table S19: L. asaccharolyticus differential abundance analysis in 66 studies and 25 disease types
Table S20: Plasma metabolomic analysis in MLVS cohort
Table S21: Module assignments and priority scores of coffee-related metabolites in L. asaccharolyticus (La), M. coli (Mc), D. formicigenerans (Df), and R. hominis (Rh)
Table S22: Interaction model L. asaccharolyticus/coffee intake against caffeine metabolites

Supplementary Figures Legends

Extended Data Figure 1. Coffee intake estimated via FFQ in four ZOE PREDICT and the MBS-MLVS cohorts. X-axis shows the estimated grams per day (ZOE PREDICT) and cups per day (MBS-MLVS), Y-axis shows the number of samples. Brown dashed lines indicate the 24.94^{th} and 88.95^{th} percentiles that correspond to the thresholds of 20 and 600 grams per day in the PREDICT1 cohort and have been used to determine the three categories never, moderate, and high coffee drinkers in the five cohorts. Continuous darker line marks the Gaussian kernel density estimation. Number of microbiome samples per cohort per category are also reported.

Extended Data Figure 2. Cross validation and Leave-one-dataset-out validation. a) receiver operating characteristic (ROC) curves and areas under the curve (AUCs) of random forest algorithms discriminating participants of the three combinations: never vs. moderate (light green), moderate vs. high (dark cyan), and high vs. never (brown), using the microbiome composition estimated by MetaPhlAn 4, assessed in a ten-fold, ten-times repeated cross-validations. **b)** ROC curve and AUCs of the same algorithm and experiment, using a leave-one-dataset-set-out (LODO) approach. Shaded areas represent the 95% confidence intervals of a linear interpolation over all the folds of the test.

Extended Data Figure 3. Microbiome-correlated variables, sex, age, BMI, and α-diversity are correlated with coffee intake. Point-biserial correlation between sex and coffee, and Spearman's correlation between age, BMI, α-diversity and coffee consumption. Correlations coefficients are then

pooled in a random-effects meta-analysis. Blue marks single study or pooled correlation which $p \geq 0.05$, red marks single/pooled correlations which $p < 0.05$.

Extended Data Figure 4. Meta-analysis of rank correlation between SGBs and coffee intake. a) Prevalences in the five cohorts considered in this study of the 40 strongest SGB-abundance and coffee-intake pooled partial Spearman's correlations found at $q < 0.001$. **b)** Single-study partial Spearman's correlation between SGB relative abundance and per-individuals total coffee intake, adjusting by age, sex, and BMI (black symbols) and pooled partial correlation (light blue markers). **c)** Single-study partial Spearman's correlation between SGB relative abundance and per-individuals caffeinated coffee intake only (black symbols), adjusting by age, sex, BMI, and decaffeinated coffee intake and pooled partial correlation (dark blue markers). Only the PREDICT1 and PREDICT3 22UKA cohorts were used in this analysis.

Extended Data Figure 5. Coffee-associated SGBs tend to be correlated in abundance with *L. asaccharolyticus*. (X-axis) correlation coefficients from the partial correlation meta-analysis with coffee consumption (**Tab. S7**) vs correlation coefficients from a correlation meta-analysis with *L. asaccharolyticus* abundance (Y-axis, **Tab. S10**). Correlations between *L. asaccharolyticus* and the other SGBs (Spearman's) were computed after centred log-ratio transformation (CLR) following zero imputation with a multiplicative replacement method to control for the problem of relative abundances' correlated structure. SGBs below 10% prevalence and not present in at least two cohorts were excluded (in total 707 SGBs were evaluated).

Extended Data Figure 6. Different styles of urban and rural surroundings do not impact *L. asaccharolyticus*-coffee association. *L. asaccharolyticus* relative abundance in never, moderate, and high coffee-drinkers and ten different rural/urban contexts that were available in the PREDICT3 UK22A cohort (n=11,547). Boxes represent the median and interquartile range (IQR) of the distributions; top and bottom whiskers mark the point at 1.5 IQR.

Extended Data Figure 7. *L. asaccharolyticus* in relation with sequencing depth and in 35 disease types. a) *L. asaccharolyticus* is highly prevalent in patients from 35 diseases from 89 combinations of study + country + disease, when prevalence across same-disease datasets was computed via random-effect meta-analysis. **b)** Standardized mean difference of arcsin-square rooted abundances of *L. asaccharolyticus* in public case-control settings from 25 disease-types shows no strong association between *L. asaccharolyticus* and diseases. Effect-sizes of the same-disease datasets were computed via random effect meta-analysis. Total sample sizes are available in **Tab. S18,19**.

Extended Data Figure 8. Unannotated metabolites covarying with trigonelline and caffeine in a *L. asaccharolyticus* dependent manner. a) Heatmap showing the standardized abundance of 15 plasma metabolites in the MBS cohort (n=213) showing the highest MACARRoN priority score for the presence of *L. asaccharolyticus*. **b)** MACARRoN priority scores for these metabolites. Samples are reported by coffee intake category. **c)** Mean scaled (z-score across metabolomes) relative abundances of the 25 metabolites in metabolomes that belong to one of the nine categories in the MLVS cohort. Enrichment of these metabolites is most prominent in *L. asaccharolyticus* and simultaneously linked to both higher coffee consumption and relative abundance (RA) of the species (Absent: RA<0.01%; Low: 0.1%>RA≥0.01%; High: RA>0.1%). For the non coffee-associated SGBs, enrichment is seen to be linked only to higher coffee intake. **d)** Clustering dendrogram of the MACARRoN between metabolites correlations. **e)** Quinic acid structure and mass-to-charge ratio suggests that six unannotated metabolites prioritized by MACARRoN are derived by modification of quinic acid.

Extended Data Figure 9. Metatranscriptomics of MLVS. a) *L. asaccharolyticus* transcripts are detected in only twelve samples by metatranscriptomics. **b)** SGB-level profiling reveals that a large majority of samples contain *L. asaccharolyticus* (SGB15154) transcripts however transcript coverage is low. **c)** Species abundance and transcript detection are correlated where transcripts from higher

abundance species are detected in a larger number of samples compared to transcripts from lower abundance species. Th red dot indicates *L. asaccharolyticus*.
