# Supplementary material for: Coffee consumption is associated with intestinal Lawsonibacter asaccharolyticus abundance and prevalence across multiple cohorts
Source: Nat Microbiol. 2024 Nov 18;9(12):3120–34. doi: 10.1038/s41564-024-01858-9 (PMC11602726; doi:10.1038/s41564-024-01858-9)
Supplement: Supplementary file 2 — Reporting Summary [file 41564_2024_1858_MOESM2_ESM.pdf]

nature portfolio

Corresponding author(s): Prof. Nicola Segata

Last updated by author(s): Paolo Manghi

Reporting Summary

Nature Portfolio wishes to improve the reproducibility of the work that we publish. This form provides structure for consistency and transparency in reporting. For further information on Nature Portfolio policies, see our Editorial Policies and the Editorial Policy Checklist.

Statistics

For all statistical analyses, confirm that the following items are present in the figure legend, table legend, main text, or Methods section.

n/a	Confirmed	
☐	☒	The exact sample size (*n*) for each experimental group/condition, given as a discrete number and unit of measurement
☐	☒	A statement on whether measurements were taken from distinct samples or whether the same sample was measured repeatedly
☐	☒	The statistical test(s) used AND whether they are one- or two-sided<br>*Only common tests should be described solely by name; describe more complex techniques in the Methods section.*
☐	☒	A description of all covariates tested
☐	☒	A description of any assumptions or corrections, such as tests of normality and adjustment for multiple comparisons
☐	☒	A full description of the statistical parameters including central tendency (e.g. means) or other basic estimates (e.g. regression coefficient) AND variation (e.g. standard deviation) or associated estimates of uncertainty (e.g. confidence intervals)
☐	☒	For null hypothesis testing, the test statistic (e.g. *F*, *t*, *r*) with confidence intervals, effect sizes, degrees of freedom and *P* value noted<br>*Give P values as exact values whenever suitable.*
☒	☐	For Bayesian analysis, information on the choice of priors and Markov chain Monte Carlo settings
☐	☒	For hierarchical and complex designs, identification of the appropriate level for tests and full reporting of outcomes
☐	☒	Estimates of effect sizes (e.g. Cohen's *d*, Pearson's *r*), indicating how they were calculated

Our web collection on statistics for biologists contains articles on many of the points above.

Software and code

Policy information about availability of computer code

Data collection	No software was used to collect the metagenomics and metabolomics data.
Data analysis	preprocessing (https://github.com/SegataLab/preprocessing), KneaData 0.3 (http://huttenhower.sph.harvard.edu/kneaddata), MetaPhlAn (version 4.0.5) (https://github.com/biobakery/MetaPhlAn/), metAML (https://github.com/segatalab/metaml) (ver. 1.1) , curatedMetagenomicDataAnalyses (https://github.com/waldronlab/curatedMetagenomicDataAnalyses), curatedMetagenomicData 3 (https://waldronlab.io/curatedMetagenomicData/), MACARRoN (https://github.com/biobakery/Macarron), HUMAnN 3.6 (https://github.com/biobakery/humann).

For manuscripts utilizing custom algorithms or software that are central to the research but not yet described in published literature, software must be made available to editors and reviewers. We strongly encourage code deposition in a community repository (e.g. GitHub). See the Nature Portfolio guidelines for submitting code & software for further information.

Data

Policy information about availability of data

All manuscripts must include a data availability statement. This statement should provide the following information, where applicable:

- Accession codes, unique identifiers, or web links for publicly available datasets
- A description of any restrictions on data availability
- For clinical datasets or third party data, please ensure that the statement adheres to our policy

Raw metagenomic samples are provided for all participants of the ZOE PREDICT Studies. Specifically, PREDICT 1 is made available as reported previously 24, whereas PREDICT 2 and PREDICT 3 US21, US22A, and UK22A cohorts are deposited in EBI under accession numbers PRJEB75460, PRJEB75462, PRJEB75463, and PRJEB75464. Sex, age, BMI, country, and the quantitative taxonomic profiles are available for each sample within the curatedMetagenomicData package 38. Raw metagenomics and metatranscriptomics reads for the MLVS cohort are deposited at NCBI under accession PRJNA354235. Data from the Health Professionals Follow-up Study, including metadata not included in the current manuscript but collected as part of the MLVS, can be obtained through written application. Raw sequencing reads and covariates for the MBS cohort are available at <BIOM-Mass link>. Further information on the cohort is available at https://www.nurseshealthstudy.org/researchers.

Research involving human participants, their data, or biological material

Policy information about studies with human participants or human data. See also policy information about sex, gender (identity/presentation), and sexual orientation and race, ethnicity and racism.

Reporting on sex and gender	We found that coffee is on average consummed more among US and UK males. As such, we peformed analyses by taking into account sex as a binary variable in which male == 1, female == 0, so that the correlations identified were robust to the greater coffee consumption among males. Metabolomics models were based on either MBS or MLVS cohorts, which are sex-specific, thus the metabolomics-related linear models were not adjusted for sex as this is implicit in the study design.
Reporting on race, ethnicity, or other socially relevant groupings	Ethnicity was not considered in this work as normally done for metagenomic studies in which environmental exposures determine the largest variability. Country was taken into account when performing meta-analysis on country-specific datasets. A total of 20 studies were about individuals named non-westernized according to a wide panel of publications which adopt the same classification and are reported in the manuscript. These individuals belong to different ethnicities which are, by definition, linked to their country.<br>Urban vs rural context of UK participants is used in one analysis. Another analysis considers the coffee intake of participants in the different macro-regions of the US. All these group numbers are reported in the supplementary materials.
Population characteristics	Age of the participants in this study is taken into account in all analyses as covariate. All participants are adults, except for one analysis which considers babies and children for the specific reason of being such. Participants age-range is reported in the Methods. For non-westernized samples, see ABOVE. 37 samples are metagenomic samples from archeological specimen. 201 are samples from non-human primates. Samples from diseased individuals were assigned to a health-related condition during the development of the resource curatedMetagenomicData, which exploits information available in the original publications including the corresponding age group and lifestyle of the individuals. PREDICT 1 includes 1,098 participants aged 18-66, (792 females [72%], 5.8x10e10 reads in total) from UK. PREDICT2 (n=975, individuals from the US aged 18-84 years, 703 females [72%], 5.8x10e10 reads sequenced in total); PREDICT3 US21 (n=11,798, aged 18-87, US, 10,270 females [87%], 4.4x10e11 reads in total); PREDICT3 US22A (n=8,470 aged 18-90, US, 7,361 females [87%], 3x10e11 reads); PREDICT3 UK22A (n=12,353, aged 18-95, UK, 9,447 females [76%], 3.9x10e11 reads in total). The Men's Lifestyle Validation Study (MLVS) 30 is a sub-study of the main Health Professionals Follow-up Study (HPFS) 51 and consisted of 700 men aged 52 to 81 years who were free of coronary heart diseases, stroke, cancer (except squamous or basal cell skin cancer), and major neurological diseases. The HSPH is an ongoing prospective cohort study of 51,529 US male health professionals aged 40 to 75 years at enrollment in 1986. Similarly, Mind-Body Study (MBS), as a sub-cohort nested in the Nurses' Health Study II (NHS II) (https://nurseshealthstudy.org), adopted the same protocol for stool sample collection as in the MLVS. The NHSII is an ongoing prospective cohort study of 116,429 US female nurses aged 25-42 at enrollment in 1989. During 22013-2014, 233 women from the Mind-Body Study (MBS) were mailed stool and blood sample collection kits and 209 women returned usable stool samples.
Recruitment	MLVS is part of the Health Professionals Follow-up Study (HPFS) recruitment program. MBS is part of the Nurses' Health Study II (NHS II) (https://nurseshealthstudy.org). The ZOE PREDICT cohorts are the results of their own recruitment program which is described at 10.21203/rs.2.20798/v1
Ethics oversight	All cohorts collection procedures complied with all ethical regulations, including the Declaration of Helsinki (2013). Ethical approval of the MBS and MLVS cohorts was granted by the Institutional Review Boards of the Brigham and Women's Hospital and the Harvard T.H. Chan School of Public Health. The ZOE PREDICT cohorts were approved, in the UK, by the Research Ethics Committee and Integrated Research Application System (IRAS 236407); in the US by the Institutional Review Board: Partners Healthcare IRB 2018P002078, clin. Trial NCT03479866 (ZOE PREDICT1); IRB Pro00033432, NCT03983733 (ZOE PREDICT2), and NCT04735835 (ZOE PREDICT3). Any procedure involving individuals from all cohorts was carried out after having received written informed consent.

Note that full information on the approval of the study protocol must also be provided in the manuscript.

Field-specific reporting

Please select the one below that is the best fit for your research. If you are not sure, read the appropriate sections before making your selection.

☒ Life sciences ☐ Behavioural & social sciences ☐ Ecological, evolutionary & environmental sciences

For a reference copy of the document with all sections, see nature.com/documents/nr-reporting-summary-flat.pdf

Life sciences study design

All studies must disclose on these points even when the disclosure is negative.

Sample size	This study considers N=22,867 individuals whose consumption of coffee was surveyed. The identification of 115 positively associated SGBs with FDR-adjusted p-values in the range of 10e-20 represents itself an evidence of sufficient power considered the specific topic.
Data exclusions	128 samples from PREDICT1 and 131 samples from PREDICT2 were excluded for the absence of nutritional records related to coffee. Participants having recorded an intake of coffee > 99th percentiles in the ZOE PREDICT cohorts were also excluded as outliers. This excluded 14 more samples from PREDICT1, 9 from PREDICT2, 94 from PREDICT3 US22A, and 131 from PREDICT3 UK22A.<br>For in vitro experiments the conditions of 5g/L instant coffee, 5g/L instant decaffeinated coffee, and 1g/L brewed decaffeinated coffee for E. coli were excluded from Figure 3d due to contamination.
Replication	In vitro experiments were conducted once for each reported study design, with five technical replicates per condition in the liquid culture experiments to monitor for potential contamination during the procedure.
Randomization	not applicable: this is an observational study, i.e. individuals coffee consumptions have not been fixed by an internevtion, but have been surveyed by questionnaires and investigated as exposure; in vitro experiments involve controlled conditions and objective measurements.
Blinding	not applicable: same reason as above. We are interested in real coffee consumption as an exposure and not as a induced treatment.

Reporting for specific materials, systems and methods

We require information from authors about some types of materials, experimental systems and methods used in many studies. Here, indicate whether each material, system or method listed is relevant to your study. If you are not sure if a list item applies to your research, read the appropriate section before selecting a response.

Materials & experimental systems

n/a	Involved in the study	
☒	☐	Antibodies
☒	☐	Eukaryotic cell lines
☒	☐	Palaeontology and archaeology
☒	☐	Animals and other organisms
☐	☒	Clinical data
☒	☐	Dual use research of concern
☒	☐	Plants

Methods

n/a	Involved in the study	
☒	☐	ChIP-seq
☒	☐	Flow cytometry
☒	☐	MRI-based neuroimaging

Clinical data

Policy information about clinical studies

All manuscripts should comply with the ICMJE guidelines for publication of clinical research and a completed CONSORT checklist must be included with all submissions.

Clinical trial registration	NCT03479866, NCT03983733, NCT04735835
Study protocol	The full trial protocols for ZOE PREDICT can be accessed at: https://zoe.com/whitepapers/the-predict-program.
Data collection	For the MBS and MLVS studies we report "The Men's Lifestyle Validation Study (MLVS) 30 is a sub-study of the main Health Professionals Follow-up Study (HPFS) 57 and consisted of 700 men aged 52 to 81 years who were free of coronary heart diseases, stroke, cancer (except squamous or basal cell skin cancer), and major neurological diseases. From 2012-2013, a total of 307 men in the MLVS provided up to two pairs of self-collected stool samples from consecutive bowel movements; each pair of samples were collected 24-72 hours apart, and the two pairs were collected approximately six months apart 30. Two blood samples were drawn, 6 months apart, to coincide with the timing of the fecal sample collection. The HSPH is an ongoing prospective cohort study of 51,529 US male health professionals aged 40 to 75 years at enrollment in 1986. Similarly, Mind-Body Study (MBS), as a sub-cohort nested in the Nurses' Health Study II (NHS II) (https://nurseshealthstudy.org), adopted the same protocol for stool sample collection as in the MLVS 29. During 22013-2014, 233 women from the Mind-Body Study (MBS) were mailed stool and blood sample collection kits and 209 women returned usable stool samples."<br>For the ZOE PREDICT cohorts, we report: "We included in this study five cohorts from the ZOE PREDICT (Personalized REsponses to

DIetary Composition Trial) program 54: PREDICT 1, which includes 1,098 participants aged 18-66, (5.8x1010 reads in total) from UK and was previously published in 55; PREDICT2 (n=975, individuals from the US are aged 18-84 years, 5.8x1010 reads sequenced in total); PREDICT3 US21 (n=11,798, aged 18-87, US, 4.4x1011 reads in total); PREDICT3 US22A (n=8,470 aged 18-90, US, 3x1011 reads); PREDICT3 UK22A (n=12,353, aged 18-95, UK, 3.9x1011)." Time frames for the idividuals recruitment in the ZOE PREDICT cohorts can be found at the above website.

Outcomes	not applicable

Plants

Seed stocks	*Report on the source of all seed stocks or other plant material used. If applicable, state the seed stock centre and catalogue number. If plant specimens were collected from the field, describe the collection location, date and sampling procedures.*
Novel plant genotypes	*Describe the methods by which all novel plant genotypes were produced. This includes those generated by transgenic approaches, gene editing, chemical/radiation-based mutagenesis and hybridization. For transgenic lines, describe the transformation method, the number of independent lines analyzed and the generation upon which experiments were performed. For gene-edited lines, describe the editor used, the endogenous sequence targeted for editing, the targeting guide RNA sequence (if applicable) and how the editor was applied.*
Authentication	*Describe any authentication procedures for each seed stock used or novel genotype generated. Describe any experiments used to assess the effect of a mutation and, where applicable, how potential secondary effects (e.g. second site T-DNA insertions, mosiacism, off-target gene editing) were examined.*
